# SUMOylation of TARBP2 regulates miRNA/siRNA efficiency

**DOI:** 10.1038/ncomms9899

**Published:** 2015-11-19

**Authors:** Cheng Chen, Changhong Zhu, Jian Huang, Xian Zhao, Rong Deng, Hailong Zhang, Jinzhuo Dou, Qin Chen, Ming Xu, Haihua Yuan, Yanli Wang, Jianxiu Yu

**Affiliations:** 1 Department of Biochemistry and Molecular Cell Biology, Shanghai Key Laboratory of Tumor Microenvironment and Inflammation, Shanghai Jiao Tong University School of Medicine, Shanghai 200025, China; 2 State Key Laboratory of Oncogenes and Related Genes, Shanghai Jiao Tong University School of Medicine, Shanghai 200025, China; 3 Department of Pathophysiology, Key Laboratory of Cell Differentiation and Apoptosis of Chinese Ministry of Education, Shanghai JiaoTong University School of Medicine, Shanghai 200025, China; 4 Department of Oncology, Institute of Oncology, Shanghai 9th People's Hospital, Shanghai Jiao Tong University School of Medicine, Shanghai 200025, China

## Abstract

Small RNA-induced gene silencing is essential for post-transcriptional regulation of gene expression; however, it remains unclear how miRNA/siRNA efficiency is regulated. Here we show that TARBP2 is SUMOylated at K52, which can be enhanced by its phosphorylation. This modification can stabilize TARBP2 via repressing its K^48^-linked ubiquitination. We find that TARBP2 SUMOylation does not influence the overall production of mature miRNAs, but it regulates miRNA/siRNA efficiency. SUMOylated TARBP2 recruits Ago2 to constitute the RNA-induced silencing complex (RISC)-loading complex (RLC), and simultaneously promotes more pre-miRNAs to load into the RLC. Consequently, Ago2 is stabilized and miRNAs/siRNAs bound by TARBP2/Dicer is effectively transferred to Ago2. Thus, these processes lead to the formation of the effective RISC for RNA interference (RNAi). Collectively, our data suggest that SUMOylation of TARBP2 is required for regulating miRNA/siRNA efficiency, which is a general mechanism of miRNA/siRNA regulation.

## Abstract (web summary)

As part of the miRNA-generating machinery, TARBP2 stabilizes the RNA-induced silencing complex (RISC) loading complex (RLC). Here, Chen *et al.* show that sumoylation of TARBP2 regulates RNAi efficiency by increasing precursor miRNAs loaded on RLC.

## Graphical abstract

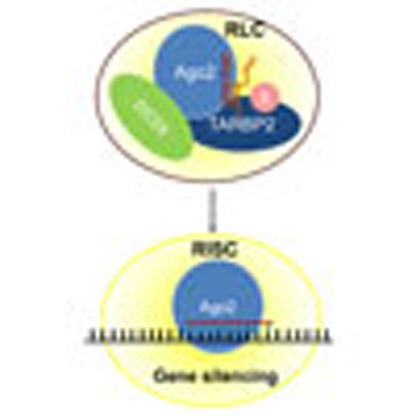

MicroRNAs (miRNAs) are a group of small noncoding RNAs that govern a number of biological processes by directly targeting messenger RNA (mRNA) transcripts^1^. Dysregulated expression of miRNAs often leads to diseases including cancer^2^,^3^. Mammalian miRNAs are ∼22-nucleotide (nt) cellular RNAs, derived from genome-encoded primary transcripts. First, these primary transcripts are processed to ∼65-nt precursor miRNAs (pre-miRNAs) by a processing complex containing ribonuclease III Drosha and double-stranded RNA-binding protein DGCR8 (refs 4, 5). Then, the stem–loop structure of pre-miRNAs can be recognized by Exportin-5/Ran-GTP on the membrane of the nucleus and transported into the cytoplasm^6^. These pre-miRNAs are substrates of miRNA-generating machinery that are composed of ribonuclease III Dicer and TARBP2 (HIV TAR RNA-binding protein) in human or LOQS in flies^7^,^8^,^9^. TARBP2 has two double-stranded RNA-binding domains (dsRBD) and one Medipal domain with mediating protein–protein interactions^10^. By directly binding to Dicer and PACT through the Medipal domain of the C terminal, TARBP2 can stabilize the RNA-induced silencing complex (RISC)-loading complex (RLC), which is composed of Dicer, TARBP2 and Argonaute2 (Ago2), for miRNA processing and gene silencing^7^,^11^,^12^,^13^,^14^. It has been reported in sporadic and hereditary cancer that TARBP2 has a frameshifted mutation, which results in instability of Dicer and eventually the defect of miRNA processing^15^.

miRNA biogenesis is strictly controlled at several levels, such as transcription, processing, itself modification and decay^16^. Post-translational modifications of the key proteins for miRNA biogenesis can also regulate miRNA biogenesis, for examples, phosphorylation on Drosha at Ser300/Ser302 maintains its nuclear localization^17^ and deacetylation of DGCR8 by HDAC1 increases its affinity with pri-miRNAs^18^. MAPK/ERK-mediated phosphorylation of TARBP2 can enhance growth-promoting miRNA production by increasing the stability of miRNA-generating complex^19^. Phosphorylation at Tyr393 of Ago2, as a key component of RISC, reduces its binding with Dicer and miRNA loading, thus inhibiting miRNA maturation and miRNA-guided gene silencing^20^,^21^. These studies suggest that post-translational modifications play important roles in regulating miRNA biogenesis and RNA-induced gene silencing.

SUMO (small ubiquitin-related modifier) is a class of ∼10-kDa polypeptide and it can be conjugated with thousands of substrates by reversible covalence. SUMOylation is an important modification^22^, which is involved in transcriptional regulation, nuclear transport, maintaining genome integrity as well as signal transduction^23^. SUMO can also non-covalently interact with the SUMO-interacting motifs (SIMs) of target proteins, whose consensus sequence contains a hydrophobic core^24^. One study reported that smoking can increase the SUMOylation level of Dicer, which may promote protein degradation and lead to the defect of miRNA processing in macrophages^25^. Most recently, we found that SUMOylation of DGCR8 at K707 controls direct function of primary miRNA^26^. Therefore, these suggest that SUMOylation may regulate biogenesis and function of miRNAs.

Here we found that TARBP2 was SUMOylated at lysine 52 (K52). SUMOylation of TARBP2 appeared not to affect mature miRNA biogenesis, but it controlled miRNA/short interfering RNA (siRNA) efficiency. SUMOylation of TARBP2 significantly promoted its binding with pre-miRNAs, and also enhanced its binding with Ago2 via SUMO1 (conjugated to TARBP2) directly interacting with SIMs of Ago2, as well as Ago2 stability. In addition, we found that TARBP2 SUMOylation was linked to tumorigenesis.

## Results

### TARBP2 is SUMOylated *in vitro* and *in vivo*

**Figure 1:**
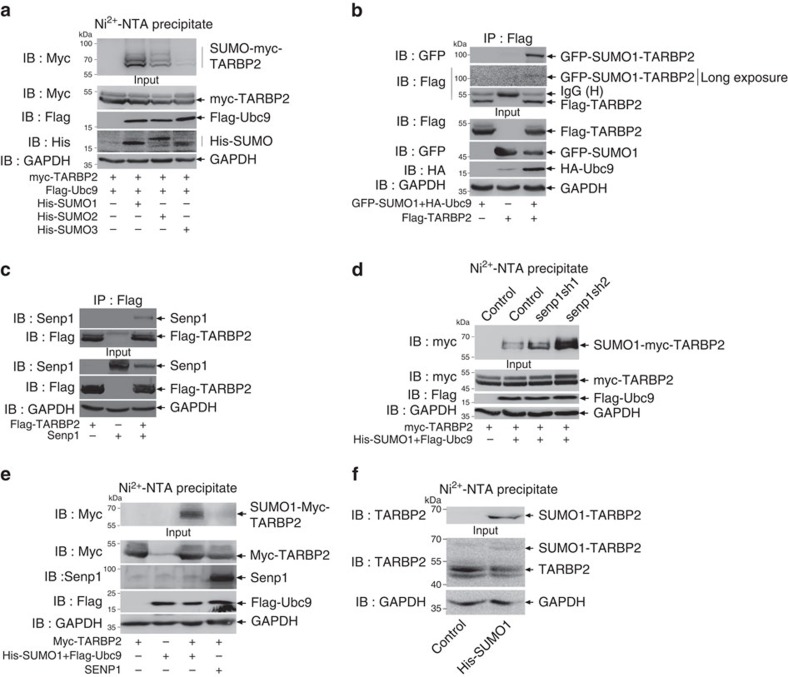
TARBP2 is SUMOylated *in vitro* and *in vivo.* (**a**) TARBP2 is mainly modified by SUMO1 in 293T cells. 293 T cells were co-transfected with myc-TARBP2, Flag-Ubc9 and His-SUMO1, or SUMO2, or SUMO3. Cell were lysed for precipitation with Ni^2+^-NTA resin and then analysed using western blot analysis with indicated antibodies. (**b**) SUMO1 conjugated covalently to TARBP2. 293T cells were transfected with indicated plasmids. Cell lysates were used forIP with anti-Flag antibody, followed by western blot analysis with anti-GFP antibody to detect the SUMOylated band. The same membrane was re-immunoblotted with anti-Flag antibody after stripping. (**c**) Senp1 interacts with TARBP2. 293T cells were transfected with indicated plasmids. Cell lysates were co-immunoprecipitated with anti-Flag antibody and were followed by western blotting with anti-Flag and anti-Senp1 antibodies. (**d**) Knockdown of Senp1 promotes TARBP2 SUMOylation. Senp1 was stably knocked down by lentiviral system carrying *Senp1* shRNAs in 293T cells (293T senp1sh1 and senp1sh2). The two stable cell lines were transfected with indicated plasmids for 48 h, and cells were lysed for Ni^2+^-NTA resin precipitation. Western blot analysis was performed to detect the levels of TARBP2 SUMOylation with anti-myc antibody. (**e**) Overexpression of Senp1 removes SUMOylation of TARBP2. Myc-TARBP2, His-SUMO1, Flag-Ubc9, with or without SENP1 were co-transfected into 293T cells. Ni^2+^-NTA resin pull down was performed to detect the TARBP2 SUMOylation level. (**f**) Endogenous TARBP2 can be SUMOylated in HeLa cells stably overexpressing His-SUMO1. Cell lysates were used for Ni^2+^-NTA resin pull down to detect the band of SUMOylated TARBP2. For full scans of western blots (**b**,**d**–**f**), see Supplementary Fig. 8.

Three major paralogues SUMO1, SUMO2 and SUMO3 are expressed in mammals^22^. To identify whether TARBP2 can be SUMOylated in cells, we transfected three different His-tagged SUMO plasmids with SUMO-conjugating enzyme E2 Flag-Ubc9 and myc-tagged TARBP2 into 293T cells, respectively (Fig. 1a). His-SUMO-conjugated TARBP2 was pulled down by using the method of Ni^2+^-NTA resin precipitation^27^,^28^. The result showed that TARBP2 was modified by all these three SUMO proteins. Among these, SUMO1 modification was much stronger than the other two. Next, we used the method of immunoprecipitation (IP) to confirm whether SUMO1 can be covalently conjugated to TARBP2. Flag-TARBP2 with or without GFP-SUMO1 and HA-Ubc9 were co-transfected into 293T cells, and then lysates were used for IP with anti-Flag antibody and immunoblotted with anti-GFP antibody, showing a specific band in a size of ∼100 kDa shifted from 50 kDa (Fig. 1b), which demonstrated that Flag-TARBP2 was conjugated with GFP-SUMO1. As Senp1 is an SUMO1 modification-specific protease^29^, we wondered whether Senp1 can bind to TARBP2 to remove the SUMO1 modification. To validate this, we performed IP of lysates from 293T cells co-transfected with Flag-TARBP2 with or without Senp1, and showed that TARBP2 indeed interacted with Senp1 (Fig. 1c). We also co-transfected myc-TARBP2 with His-SUMO1/Flag-Ubc9 into the stable Senp1-knockdown 293T cell lines (Supplementary Fig. 1) and conducted the SUMOylation assays, revealing that TARBP2 SUMOylation was enhanced with varying degrees in the Senp1-knockdown cell lines (Fig. 1d). However, overexpressing Senp1 in 293T cells even with His-SUMO1/Flag-Ubc9 removed the SUMO1 modification-specific band of TARBP2 (Fig. 1e). Furthermore, we generated HeLa cell line stably overexpressing His-SUMO1 and found that endogenous His-SUMO1 modified TARBP2 could be pulled down by Ni^2+^-NTA resin (Fig. 1f). Therefore, above data demonstrate that TARBP2 is modified by SUMO1 in cells.

### K52 is the main SUMO site of TARBP2

**Figure 2:**
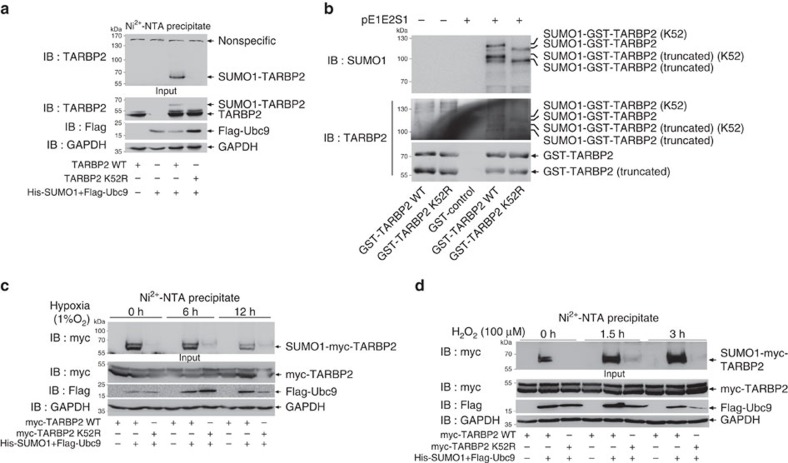
TARBP2 is mainly SUMOylated at K52. (**a**) Mutation K52R abolishes TARBP2 SUMOylation in 293T cells. pLPC-TARBP2-WT or -K52R with His-SUMO1/Flag-Ubc9 was co-transfected into 293T cells. Forty-eight hours after transfection, cells were lysed and Ni^2+^-NTA pull down was performed to detect TARBP2 SUMOylation. (**b**) Mutation K52R impairs TARBP2 SUMOylation in an *E. coli* system. Plasmid pGEX4T1-TARBP2-WT or -K52R with pE1E2S1 plasmid were co-transfected into *E. coli* BL21 (DE3). Western blot analysis was conducted with anti-SUMO1 antibody after GST pull down, and the same membrane was also detected with anti-TARBP2 antibody after stripping. (**c**) Hypoxia downregulates SUMOylation of TARBP2. 293T cells transfected with indicated plasmids were cultured under 1% of oxygen condition (hypoxia) for indicated time before being harvested. Ni^2+^-NTA resin pull down was performed to detect SUMOylated TARBP2. (**d**) Hydrogen peroxide (H_2_O_2_) upregulates SUMOylation of TARBP2. 293T cells transfected with indicated plasmids were treated with 100 μM of H_2_O_2_ for indicated time before being harvested. Ni^2+^-NTA resin pull down was performed to detect SUMOylated TARBP2. For full scans of western blots (**a**–**d**), see Supplementary Fig. 8.

According to the prediction made by the SUMOPlot software, TARBP2 has several putative SUMO sites (Supplementary Table 1). Among those candidates, L^51^KAE^54^ with the conserved SUMO-motif sequence of ΨKXD/E has the highest possibility score; therefore, we mutated the lysine 52 into arginine (K52R). The SUMOylation assay revealed that the mutation K52R almost completely abolished TARBP2 SUMOylation (Fig. 2a), indicating that TARBP2 SUMOylation mainly occurs at K52. To confirm this, we also introduced a prokaryotic SUMOylation assay in *E. coli* BL21 with a plasmid pE1E2S1 co-expressing two enzymes E1 and E2 and SUMO1 refs 30, 31). Purified unSUMOylated GST (glutathione *S*-transferase)-TARBP2 from transfected even with or without pE1E2S1 showed an additional band of size ∼60 kDa, supposedly a truncated form, except the band of 75-kDa as expected size (Fig. 2b, bottom panel). After GST pulldown, immunoblotting with anti-SUMO1 antibody showed that GST-TARBP2-WT or -K52R in those co-transfected with pE1E2S1 (Lanes 4 and 5) was SUMOylated when compared with those transfected without pE1E2S1 (Lanes 1, 2; Fig. 2b, upper panel). These bands were also confirmed SUMOylated GST-TARBP1 by detection with anti-TARBP2 antibody on the same membrane after stripping (Fig. 2b, middle panel). Four specific bands with sizes of ∼90∼120 kDa were detected in GST-TARBP2-WT, whereas two of the four bands as indicated disappeared in GST-TARBP2-K52R. We considered that the lower two of the four bands that were detected by anti-SUMO1 antibody were SUMOylated GST-TARBP2-truncated forms. From these assays, we observed other two SUMOylation bands that still existed in the K52R mutant, indicating that TARBP2 probably has other potential SUMO sites. Nevertheless, in eukaryotic cells K52 seemed to be the only SUMO site.

Moreover, we found that the SUMOylation levels of TARBP2 could be regulated by oxidative stress. At 36 h after transfection, 293T cells were treated with hypoxia (1% O_2_) for 0, 6 and 12 h, and then the SUMOylation assays were performed. The result showed that SUMOylation of TARBP2 gradually decreased at 6 and 12 h when compared with 0 h (untreated; Fig. 2c). More interestingly, in contrast to hypoxia, the treatment with 100 μM hydrogen peroxide (H_2_O_2_) very significantly enhanced the SUMOylation levels of TARBP2 at 1.5 and 3 h (Fig. 2d). However, the mutant TARBP2 K52R failed to response to both of the above stimulations. These data strongly support that K52 is the only SUMO site of TARBP2 in eukaryotic cells.

### Phosphorylation of TARBP2 enhances its SUMOylation

**Figure 3:**
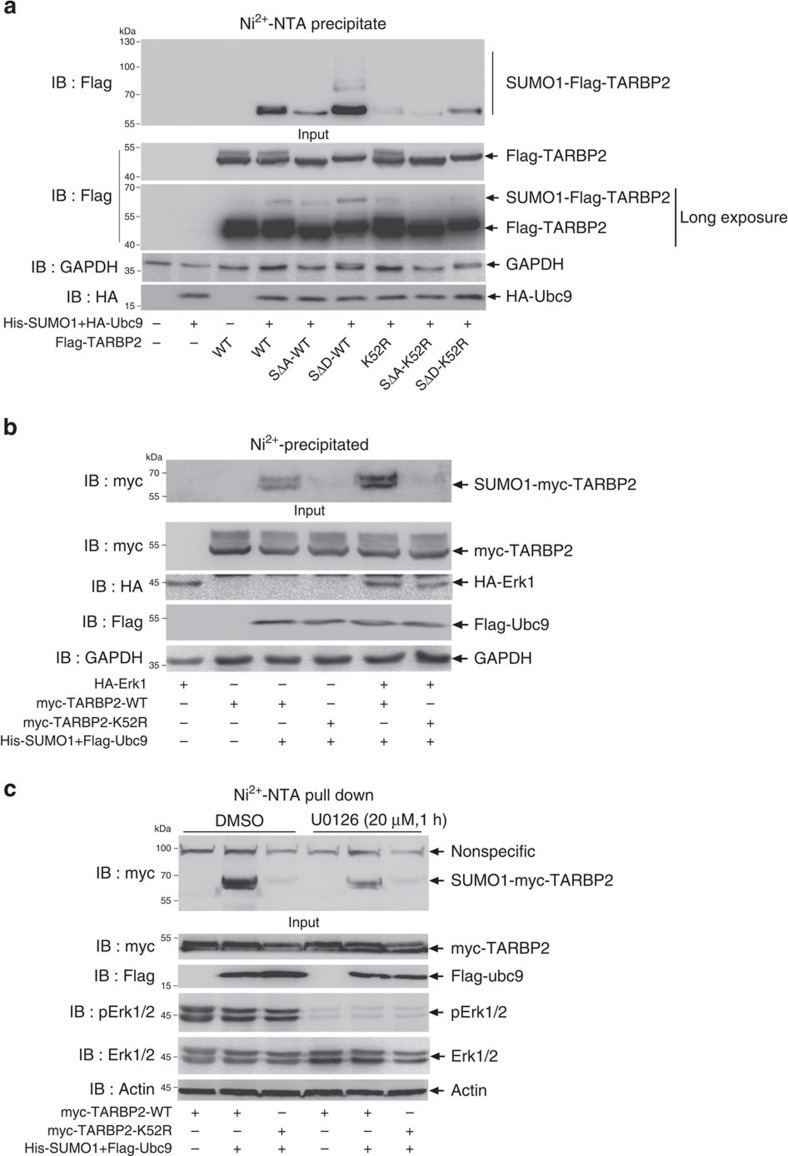
Phosphorylation promotes SUMOylation of TARBP2. (**a**) Phosphorylation of TARBP2 enhances its SUMOylation. 293T cells were transfected with indicated plasmids, and SUMOylation assays by Ni^2+^-NTA resin pull down were conducted as before. ‘WT' means K52 is not mutated; ‘SΔA' represents TARBP2 phosphomutant (Serines142/152/283/286-to-Alanines); and ‘SΔD' represents TARBP2 phosphomimic (Serines142/152/283/286-to-Aspartates). (**b**) Overexpression of Erk increases the SUMOylation level of TARBP2. 293T cells were co-transfected with indicated plasmids, and the SUMOylation assay by Ni^2+^-NTA resin pull down was conducted as before. (**c**) Repression of Erk activity by the inhibitor U0126 reduces TARBP2 SUMOylation. 293T cells were transfected with indicated plasmids. Cells were treated with U0126 (20 μM) for 1 h before being harvested. The SUMOylation assay by Ni^2+^-NTA resin pull down was conducted as before. For full scans of western blots (**a**–**c**), see Supplementary Fig. 8.

Since phosphorylation can promote SUMOylation^32^,^33^,^34^ and TARBP2 phosphorylation by MAPK/Erk enhances its stability^19^, it is possible that phosphorylation might also regulate TARBP2 SUMOylation. As there are four major sites of phosphorylation by the MAPK/Erk pathway, S142, S152, S283 and S286 (Supplementary Fig. 2), we mutated all these four serines to alanine (SΔA) for mimicking the unphosphorylated state or to aspartates (SΔD) for mimicking the phosphorylated state, and generated four constructs TARBP2-SΔA-WT, TARBP2-SΔD-WT, TARBP2-SΔD-K52R and TARBP2-SΔA-K52R, and transfected them with His-SUMO1/Flag-Ubc9 into 293T cells, respectively (Fig. 3a). The SUMOylation assays showed that TARBP2-SΔD-WT strongly enhanced TARBP2 SUMOylation (Fig. 3a, lane 6) compared with that of TARBP2-WT (lane 4), whereas SUMOylation of TARBP2-SΔA-WT was markedly diminished (lane 5). On the contrary, the three TARBP2-K52R constructs with either SΔA or SΔD had the same tendency that the SUMOylation levels were greatly removed (lanes 7–9). Moreover, these data indicate that phosphorylation by MAPK/Erk enhances SUMOylation of TARBP2. Indeed, Erk1 notably increased the SUMOylation level of TARBP2-WT (Fig. 3b, lane 5 compared with lane 3), while SUMOylation of TARBP2-K52R was still not easily observed. Moreover, the reduced phosphorylation levels of Erk by an inhibitor U0126 significantly weakened TARBP2 SUMOylation (Fig. 3c).

### SUMOylation of TARBP2 inhibits its ubiquitination

**Figure 4:**
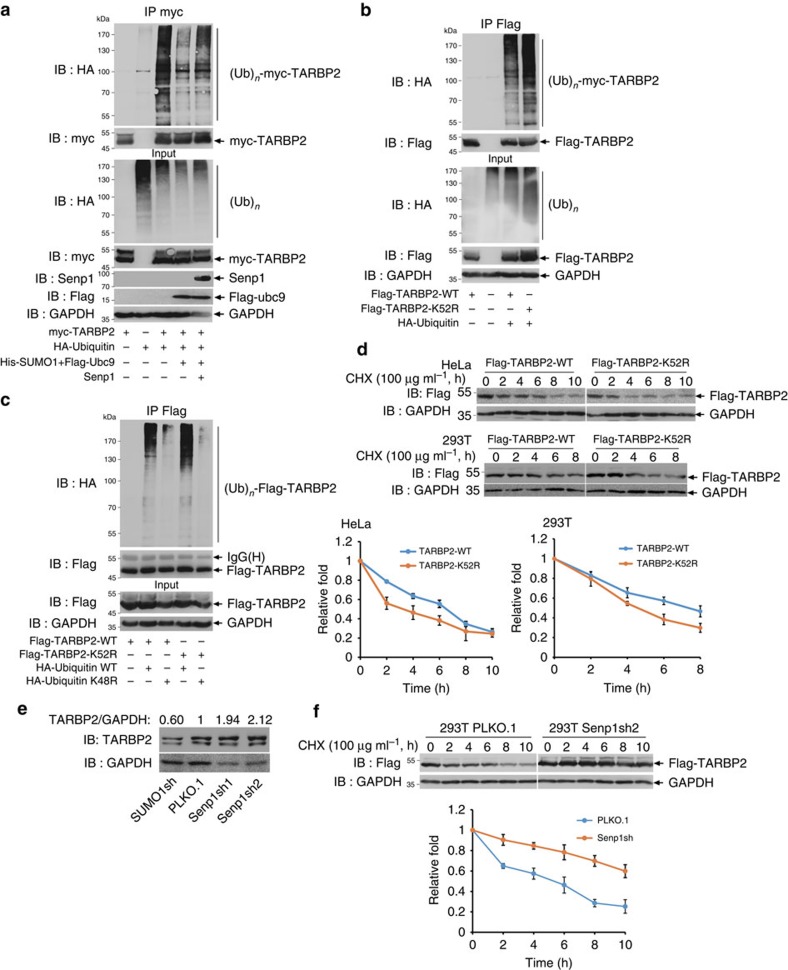
SUMOylation stabilizes TARBP2 by reducing ubiquitination. (**a**) SUMOylation of TARBP2 reduces its ubiquitination. 293T cells transfected with indicated plasmids were lysed with RIPA buffer. Cell lysates were used for IP with anti-myc antibody and followed by western blot analysis with anti-HA antibody to detect ubiquitination of TARBP2. Lysates as an Input were also immunoblotted with anti-myc, anti-Flag, anti-HA and anti-GAPDH antibodies. (**b**) SUMOylation of TARBP2 represses its ubiquitination. 293T cells were transfected with HA-Ubiquitin and Flag-TARBP2-WT or -K52R. IP with anti-Flag antibody and western blot analysis were performed as before. (**c**) SUMOylation of TARBP2 mainly inhibits K48-linked ubiquitination. 293T cells transfected with Flag-TARBP2 and HA-Ubiquitin were lysed and used for IP with anti-Flag antibody. K48R indicated HA-Ubiquitin that bore a mutation on Lysine48 to Arginine. (**d**) SUMOylation of TARBP2 increases its stability. HeLa and 293T cell lines stably expressing Flag-TARBP2-WT or -K52R were seeded at the same cell density and cultured for 24 h before the treatment with CHX (100 μg ml^−1^) with indicated time course. Cell lysates were analysed using western blot analysis with anti-Flag and anti-GAPDH antibodies. The line charts presented for the relative fold of TARBP2 were analysed using ImageJ. (**e**) The SUMOylation level is positively corrected with the expression of TARBP2. Stable Senp1 or SUMO1 knock down 293T cell lines were generated and the expression levels of endogenous TARBP2 in these cell lines were analysed using western blot analysis with anti-TARBP2 antibody. (**f**) TARBP2 has a longer half-life under higher SUMOylation level. Stable Senp1 knock down and control 293T cell lines were treated with CHX (100 μg ml^−1^) with indicated time course. Cell lysates were analysed using western blot analysis. The line charts presented for the relative fold of TARBP2 are analysed using ImageJ. For full scans of western blots (**a**,**b**,**d**), see Supplementary Fig. 8.

Since SUMOylation can regulate the stability of its target proteins^33^,^35^, and TARBP2 phosphorylation can increase its own stability thereby enhancing the formation of miRNA-generating complex^19^, we wanted to verify whether SUMOylation of TARBP2 controls its stability. To detect whether SUMOylation affects ubiquitination of TARBP2, we co-transfected myc-tagged TARBP2, HA-tagged ubiquitin, with or without Ubc9/SUMO1. When only ubiquitin was added, TARBP2 was abundantly ubiquitinated (Fig. 4a, lane 3). However, interestingly, TARBP2 ubiquitination was markedly reduced (lane 4) in the TARBP2 co-transfected with Ubc9/SUMO1, which was partially rescued (lane 5) by co-transfection of Senp1, indicating that TARBP2 SUMOylation can decrease its ubiquitination. Thus, we questioned whether the mutation K52R may induce TARBP2 ubiquitination. As expected, the mutant TARBP2-K52R was ubiquitinated much more than TARBP2-WT (Fig. 4b). It has been reported that TARBP2 is degraded through ubiquitination modulated by Merlin^36^; therefore, we wondered whether TARBP2 degradation is exclusively mediated by K48-linked polyubiquitination. Therefore, we performed the ubiquitination assay by introducing a mutated ubiquitin (Ub-K48R), and showed that both TARBP2-WT and TARBP2-K52R were ubiquitinated (Fig. 4c, lanes 2 and 4), the latter being more strongly ubiquitinated by ubiquitin-WT. However, the ubiquitination levels were very low and there was no significant difference between TARBP2-WT and -K52R when Ub-K48R was added (lanes 3 and 5), confirming that TARBP2 is degraded via the K48-linked polyubiquitination.

To test the effect of TARBP2 SUMOylation on its stability, we generated HeLa and 293T cell lines stably expressing Flag-tagged TARBP2-WT or -K52R (Supplementary Fig. 3a) and compared their half-lives by performing a time course assay by treatment of an inhibitor cycloheximide (CHX). TARBP2-WT was more stable (*t*_1/2_∼7 h) than TARBP2-K52R, whose half-life was ∼3–4 h in HeLa and 293T cells (Fig. 4d). Next, we compared the expression levels of endogenous TARBP2 in 293T SUMO1 (Supplementary Fig. 3b) or Senp1 knockdown (Supplementary Fig. 1) cell lines, and found that TARBP2 was accumulated in Senp1 knockdown cell lines while decreased in SUMO1 knockdown cell lines (Fig. 4e). Furthermore, we tested the half-life of endogenous TARBP2 in Senp1 knockdown 293T cell lines (Fig. 4f). Consistent with above results (Fig. 4d), TARBP2 in the Senp1 knockdown cell line was more stable (*t*_1/2_∼7 h) than that in control cell line (*t*_1/2_∼4 h). These results further support that TARBP2 SUMOylation enhances the protein stability by reducing its K48-linked polyubiquitination.

### SUMOylation of TARBP2 may suppress tumour progression

**Figure 5:**
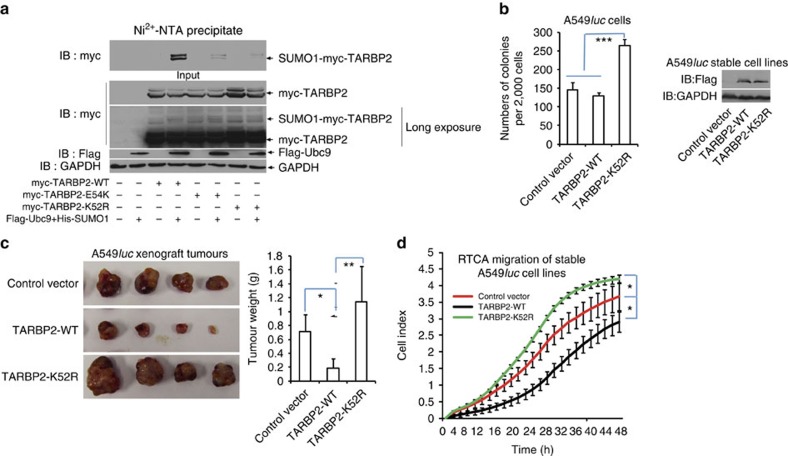
SUMOylation of TARBP2 may suppress tumour progression. (**a**) The mutation E54K abolishes SUMOylation of TARBP2. Myc-TARBP2-WT, or -K52R or E54K with Flag-Ubc9 and His-SUMO1 were co-transfected into 293T cells. The SUMOylation assay by Ni^2+^-NTA resin pull down was conducted as before. For full scans of western blot analysis, see Supplementary Fig. 8. (**b**) SUMOylation of TARBP2 suppresses anchorage-independent growth. Each of A54*9*^*luc*^ stable cell line expressing the control vector, or Flag-TARBP2-WT, or Flag-TARBP2-K52R (right panels), were seeded at a density of 2,000 cells per well in 1.5 ml of medium containing 10% FBS with 0.35% soft agarose and were layered on 0.6% solidified agarose. Photographs were taken 12 days after seeding, and the number of colonies was scored. Each value represents the mean±s.e.m. of three independent experiments with triplicates (left panels). (**c**) SUMOylation of TARBP2 suppresses xenograft tumour growth *in vivo*. Each of stable A54*9*^*luc*^ cell line expressing the control vector, or Flag-TARBP2-WT, or Flag-TARBP2-K52R (2.5 × 10^6^ cells/each) was injected subcutaneously into male BALB/c nude mice (*n*=4) individually. Mice were killed 6 weeks later, and tumours were dissected (left panels) and assessed by weight (right panels). (**d**) SUMOylation of TARBP2 inhibits tumour cell migration. The migration assay of stable A54*9*^*luc*^ cell lines was conducted with xCELLigence RTCA-DP and the kinetic curves were real-time-recorded. Differences between individual groups as indicated were analysed using the *t*-test (two-tailed and unpaired), and *P* values of <0.05 (*) or <0.01 (**) or <0.001 (***) are considered significant.

We searched somatically acquired mutations in human cancer with the database of Catalogue of Somatic Mutations in Cancer (COSMIC, Release v71). TARBP2 has 30 substitution missense mutations, accounting for 57.69% in total mutations. One of these mutations p.E54K (c.160G>A) is found in one sample of breast carcinoma (Supplementary Fig. 4a), and supposedly this mutation can disrupt SUMO1 conjugation to K52. To validate this, we generated this mutant construct and performed the SUMOylation assay, revealing that, similarly to K52R, the mutation E54K greatly abrogated SUMOylation of TARBP2 (Fig. 5a). This result suggests that SUMOylation of TARBP2 is potentially connected with tumorigenesis.

To investigate that TARBP2 SUMOylation is involved in tumorigenesis, we generated A549^*luc*^ and M12^*luc*^ stable cell lines expressing the lenti-Vector, Flag-TARBP2-WT or Flag-TARBP2-K52R with the polyclonal lentiviral infections (Fig. 5b and Supplementary Fig. 4b). The soft-agar colony formation assays were performed in the presence of fetal bovine serum (FBS) equaling to complete media of each stable cell line. Interestingly, we found that these two types of stable cell lines transfected with TARBP2-K52R remarkably increased the numbers of colonies compared with that transfected with the lenti-Vector or Flag-TARBP2-WT (Fig. 5b and Supplementary Fig. 4b), whereas that transfected with Flag-TARBP2-WT appeared to slightly decrease the growth ability compared with those in the lenti-Vector transfected ones. Moreover, we also investigated whether TARBP2 SUMOylation influences xenograft tumour growth *in vivo*. Stable A549^luc^ cell lines were inoculated subcutaneously into the back of nude mice. Bioluminescent imaging assessment was performed at 4 weeks after injection, showing that tumours in the TARBP2-K52R group were detected to be larger than those in the vector group, whereas tumours in the TARBP2-WT group were the smallest (Supplementary Fig. 4c). Tumours were weighted after killing the nude mice at 6 weeks after injection (Fig. 5c), showing the similar pattern of results as in above bioluminescent imaging. Thus, these data indicate that SUMOylation at K52 of TARBP2 inhibits anchorage-independent growth and xenograft tumour growth.

To further determine whether TARBP2 SUMOylation also influences tumour cell migration, the RTCA (real-time cell analysis) migration assay^31^ was conducted to evaluate cell motilities of stable A549^*luc*^ cell lines. Compared with the control Vector, TARBP2-K52R substantially promoted while TARBP2-WT inhibited tumour cell migration (Fig. 5d). Taken together, our data suggest that SUMOylation of TARBP2 may play roles in suppression of tumour growth^15^,^37^ and tumour cell migration.

### TARBP2 SUMOylation does not affect miRNA production

**Figure 6:**
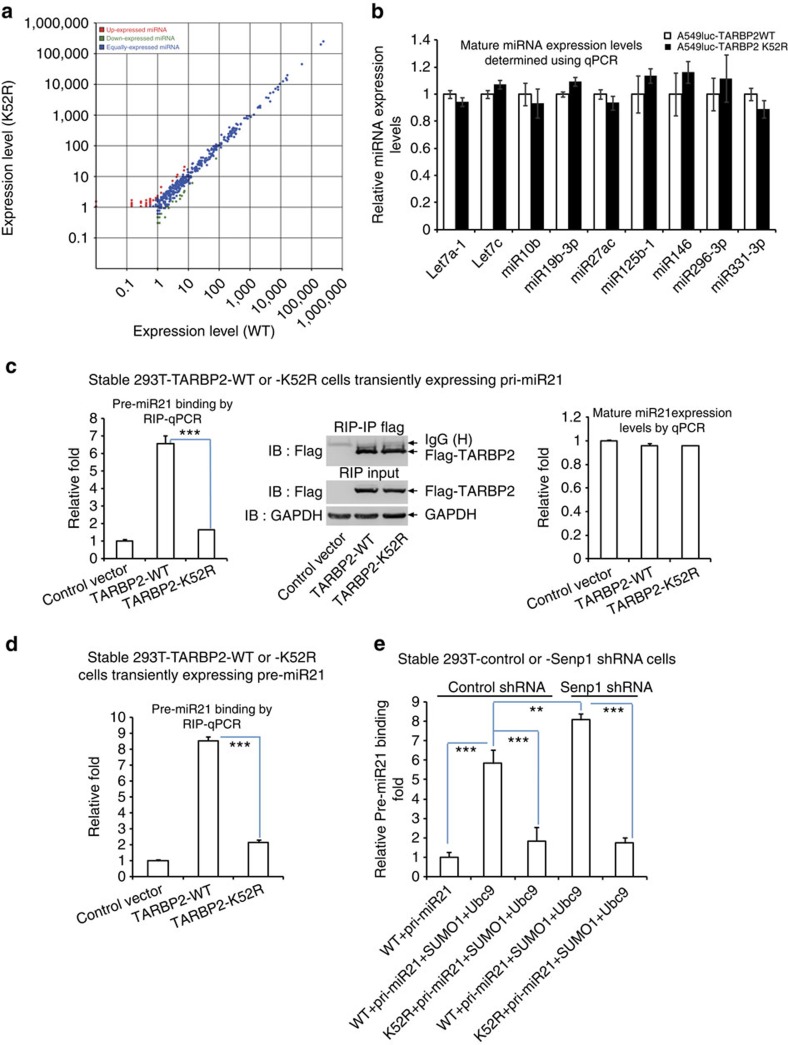
SUMOylation of TARBP2 promotes its binding with pre-miRNAs. (**a**) The mutation K52R of TARBP2 slightly influences mature microRNA production. Differential expression analysis of mature miRNA profiles was scatter-plotted based on high-throughput small RNA-sequencing data of A549^*luc*^ stable cell lines expressing Flag-TARBP2-WT (*x* axis) and -K52R (*y* axis). (**b**) The expression levels of mature miRNAs are not much different between TARBP2-WT and -K52R expressed in cells. Quantitative PCR was performed to assess the expression of let-7a-1, let-7c, miR-10b, miR-19b-3p, miR-27ac, miR125b-1, miR-146, miR-296-3p and miR-331-3p in A549^*luc*^ stable cell lines expressing Flag-TARBP2-WT and -K52R. (**c**–**e**) SUMOylation of TARBP2 enhances the binding between TARBP2 and pre-miRNAs. (**c**) Stable 293T cell lines expressing the control vector, Flag-TARBP2-WT or -K52R were transiently transfected with pri-miR21. Thirty-six hours after transfection, cells were lysed for IP with anti-Flag antibody to pull down RNAs. Bound RNAs were extracted and analysed using real-time quantitative PCR for pre-miR21. Left panel: relative fold of pre-miR21 binding with Flag-TARBP2-WT or -K52R. Middle panel: efficiency of IP was detected with western blot analysis. Right: as a control, the expression levels of mature miR21 were analysed with total RNAs using quantitative PCR. (**d**) Stable 293T cell lines expressing control vector, Flag-TARBP2-WT or -K52R were transiently transfected with pre-miR21. The RIP assay was performed as **c**. (**e**) Stable control or Senp1 shRNA knockdown 293T cell lines were transfected with indicated plasmids. Thirty-six hours after transfection, cells were lysed and the RNA-binding assay was performed. The relative fold of pre-miR125b1 binding with TARBP2 was shown with quantitative PCR. Differences between individual groups as indicated were analysed using the *t*-test (two-tailed and unpaired), and *P* values of <0.01 (**) or <0.001 (***) are considered significant.

Above data have revealed that TARBP2 SUMOylation is potentially linked to its function of inhibiting tumour progression; therefore, next we attempted to explore the underlying molecular mechanism. As the SUMO site K52 of TARBP2 is located in its dsRBD1 domain, we wanted to verify whether TARBP2 modification has an affect on the global miRNA expression profile. To this end, we used A549^*luc*^ cell lines stably expressing Flag-tagged TARBP2-WT or TARBP2-K52R for deep high-throughput sequencing according to our previous protocol^38^. Sequencing data of these two cell lines showed that the mature miRNA expression profiles were slightly but not significantly changed (Fig. 6a, Supplementary Data 1). To validate this, we performed the quantitative PCR for selected eight miRNAs and found that the expression levels of these miRNAs were indeed changed not much between TARBP2-WT and -K52R expressed in A549^*luc*^ cells (Fig. 6b). These results indicate that TARBP2 SUMOylation does not affect the production of mature miRNAs.

### SUMOylation promotes TARBP2 binding with pre-miRNAs

Next, to explore whether the mutation K52R affects the binding of TARBP2 with pre-miRNAs, we performed an RNA IP assay (RIP) by transfection of primary miRNA21 (pri-miR21, as an example here) into 293T cells stably expressing TARBP2-WT or -K52R, and then IP with anti-Flag antibody for pull-down of TARBP2-RNA complexes (Fig. 6c, middle panel). We found that TARBP2-K52R bound much less with precursor miR21 (pre-miR21; Fig. 6c, left panel) compared with TARBP2-WT, although the expression levels of the mature miR21 were not changed as expected (Fig. 6c, right panel). The similar results were observed in 293T cells transiently co-transfected with pri-miR21 and TARBP2-WT or -K52R (Supplementary Fig. 5a). Moreover, pre-miR21 and pre-miR30a were also transfected into the above stable 293T-TARBP2-WT or -K52R cells for RIP assay, showing that the binding between pre-miRNA and TARBP2-WT was much stronger than that with TARBP2-K52R (Fig. 6d and Supplementary Fig. 5b). Therefore, these data suggest that SUMOylation of TARBP2 potentially enhances the binding between TARBP2 and pre-miRNAs.

To verify this hypothesis, stable 293T cell-infected control or Senp1 short hairpin RNA (shRNA) generated by the lentiviral system were transfected with pri-miR21, TARBP2-WT or -K52R and SUMO1+Ubc9. TARBP2-WT binding with pre-miR21 was distinctly increased with about six- or eightfold by the transfection of SUMO1+Ubc9 in 293T control or Senp1 shRNA cells, respectively. However, TARBP2-K52R binding with pre-miR21 was much less as well as not affected even when SUMO1+Ubc9 was co-transfected into both control and Senp1 shRNA 293T cells (Fig. 6e). In addition, we used pri-miR125b1 to perform the similar experiment and observed the same pattern of results (Supplementary Fig. 5c).

### SUMOylation of TARBP2 stabilizes Ago2

**Figure 7:**
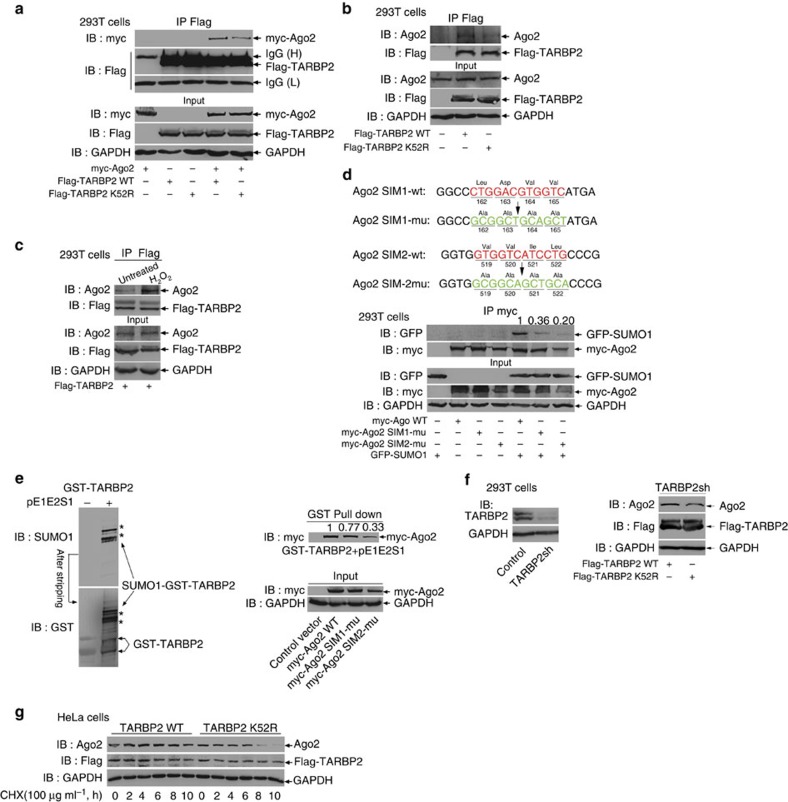
SUMOylation of TARBP2 increases its binding with Ago2. (**a**,**b**) The SUMO-site mutation K52R of TARBP2 affects the interaction between TARBP2 and Ago2. 293T cells were transfected Flag-TARBP2-WT or -K52R with (**a**) or without (**b**) myc-Ago2. Forty-eight hours after transfection, cells were lysed for IP with anti-Flag antibody, followed by western blotting with anti-Myc (**a**) or anti-Ago2 (**b**) antibody, respectively. (**c**) SUMOylation enhances TARBP2 binding with Ago2. 293T cells transfected with Flag-TARBP2 were treated with 100 μM H_2_O_2_ for 3 h before being harvested. Cell lysates were used for IP with anti-Flag antibody, followed by western blotting with anti-Myc antibody. (**d**) SUMO1 interacts with two SIMs of Ago2. Upper panels: putative SIMs of Ago2 and their mutations (SIM1-wt: ^162^L-D-V-V^165^, SIM1-mu: A-A-A-A; SIM-2-wt: ^519^V-V-I-L^522^, SIM2-mu: A-A-A-A. Lower panels: plasmid myc-Ago2-WT, -SIM1-mu or -SIM2-mu with/without GFP-SUMO1 were transfected into 293T cells. IP with anti-Myc antibody and immunoblotting with anti-GFP antibody were performed. (**e**) Ago2 interacts with SUMO1 conjugated to TARBP2 via its SIMs. pGEX4T1-TARBP2 with or without pE1E2S1 were co-transfected into *E. coli* BL21 (DE3). GST-TARBP2 protein was purified, and then immunoblotting with anti-SUMO1 and anti-GST antibodies was performed (left panels). This validated that highly SUMOylated GST-TARBP2 was used for pull down of the same amount of each lysate from 293T cells transfected with the control vector, myc-Ago2 WT or SIM1-mu or SIM2-mu, followed by immunoblotting with anti-Myc antibody (right panels). Cell lysates were also used as an Input. (**f**) The SUMO-site mutation K52R of TARBP2 decreases Ago2. Stable endogenous TARBP2-knocked down 293T cell line (namely TARBP2sh 293 T) was generated by the lentiviral system containing shRNA targeting 3′-UTR of TARBP2 mRNA (left panels). TARBP2sh 293T cells were transfected with Flag-TARBP2-WT or -K52R. Immunoblotting with anti-Dicer, anti-Ago2, anti-Flag and anti-GAPDH antibodies was performed. (**g**) SUMOylation of TARBP2 stabilizes Ago2. Stable HeLa cell lines expressing Flag-TARBP2-WT or -K52R were treated with 100 μg ml^−1^ CHX for indicated time and cell lysates were immunoblotted with anti-Ago2, anti-Flag and anti-GAPDH antibodies. For full scans of western blots (**a**,**b**,**d**,**g**), see Supplementary Fig. 8.

Next, we investigate whether SUMOylation affects the binding of TARBP2 with its known interacting proteins of the RNA-loading complex, including PKR, KSRP, PACT, DICER and AGO2. 293T cells co-transfected with Flag-TARBP2-WT or -K52R and HA-PKR or GFP-KSRP or HA-PACT or myc-AGO2, respectively, were lysed for IP with antibody anti-Flag and then immunoblotted, showing that the mutation K52R did not influence the interaction of TARBP2 with exogenous PKR, KSRP, PACT and endogenous DICER (Supplementary Fig. 6a–d). However, strikingly, we observed that the mutant TARBP2-K52R bound less either exogenous or endogenous Ago2 compared with TARBP2-WT (Fig. 7a,b). Moreover, we have shown the enhanced SUMOylation of TARBP2 by the treatment with H_2_O_2_ (Fig. 2d), which significantly increased TARBP2, binding more Ago2 (Fig. 7c).

It has been reported that SIMs can regulate the interaction between proteins^39^,^40^,^41^; therefore, we first analysed the sequence of Ago2 using the GPS-SUMO software for SIMs^42^. Taken the prediction results and consensus motifs together, we found that Ago2 has two conserved SIMs, located in amino-acid residues 162–166 and 519–523, namely SIM1 and SIM2 (Fig. 7d, upper panels). To test whether these two SIMs of Ago2 mediate its binding with SUMOylated TARBP2, we transfected Ago2-WT or Ago2-SIM1-mu or Ago2-SIM2-mu with GFP-SUMO1 into 293T cells. The result revealed that both of these two mutants reduced interaction with GFP-SUMO1 compared with Ago2-WT (Fig. 7d, lower panels). More confidently, we used purified highly SUMOylated GST-TARBP2 co-expressed with pE1E2S1 in *E. coli* to pull down the same amount of lysates from 293T cells transfected with Ago2-WT and Ago2-SIM mutants, showing a significant reduction of SUMOylated GST-TARBP2 binding with both of Ago2 SIM mutants, especially Ago2-SIM2-mu, when compared with Ago2-WT (Fig. 7e). However, the binding of GST-TARBP2 (expressed without pE1E2S1) with either Ago2-WT or two SIM mutants was comparable (Supplementary Fig. 6e). These results demonstrate that TARBP2 SUMOylation promotes Ago2 interacting with TARBP2 via its SIMs binding to SUMO1, which is conjugated to TARBP2.

To further investigate the consequence of the enhanced interaction between TARBP2 and Ago2 mediated by SUMOylation, we generated a 293T cell line whose endogenous TARBP2 was stably knocked down by shRNA targeting its 3′-untranslated repeat (UTR; Fig. 7f, left panels). Flag-TARBP2-WT or -K52R was transiently re-expressed in this cell line and endogenous Ago2 and Dicer were tested with western blot analysis. Compared with the expression of TARBP2-WT, the expression of TARBP2-K52R led to a reduction in Ago2 besides Dicer, indicating that SUMO-modified TARBP2 may stabilize Ago2 (Fig. 7f, right panels). To further verify this, stable HeLa cell lines expressing Flag-TARBP2-WT or -K52R were treated with the inhibitor CHX in a time course from 2 to 10 h, and the protein decay was analysed by immunoblotting showing that the half-life of TARBP2-K52R was shorter than that of TARBP2-WT. In addition, more interestingly, Ago2 also had a longer half-life in the stable TARBP2-WT cell lines than that in the stable TARBP2-K52R cell lines (Fig. 7g). These suggest that SUMOylation of TARBP2 is required for stabilization of Ago2 by increasing the binding between them.

### SUMOylation of TARBP2 regulates miRNA/siRNA efficiency

**Figure 8:**
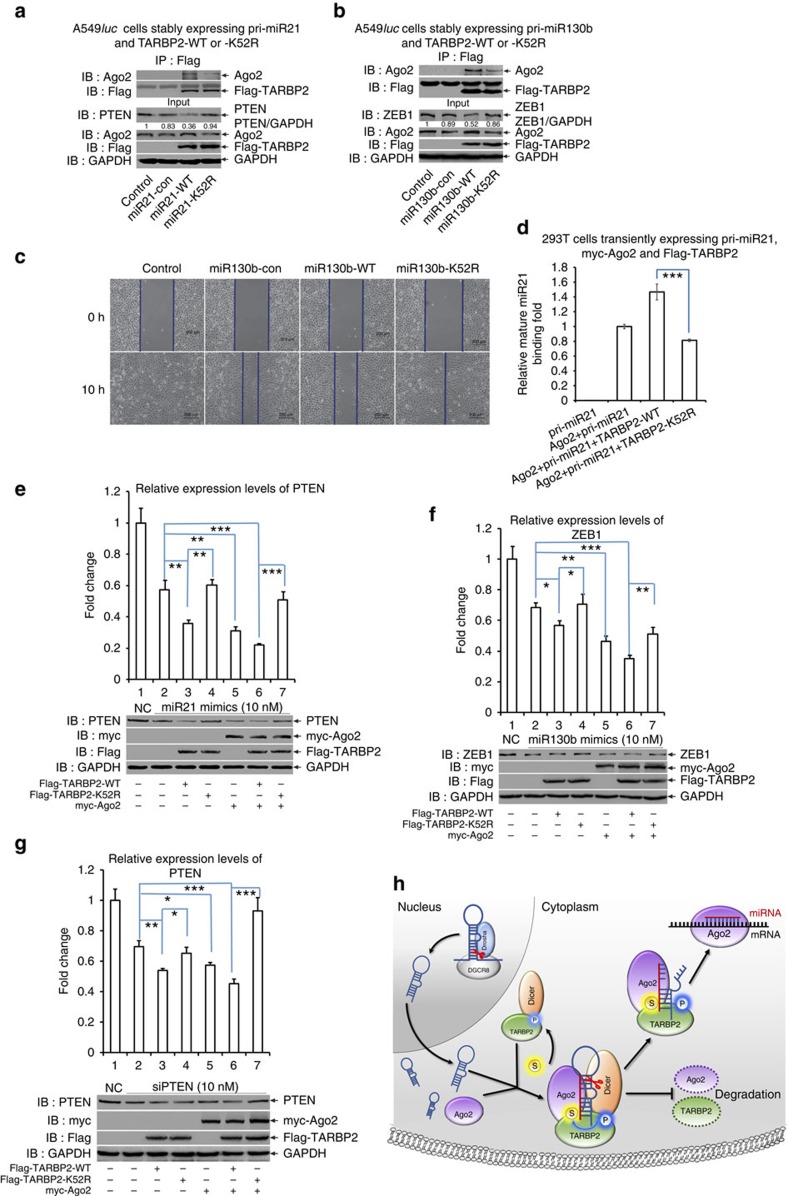
SUMOylation of TARBP2 controls miRNA/siRNA efficiency. (**a**,**b**) SUMOylation of TARBP2 is required for efficient RNA-induced gene silencing by recruiting more Ago2. A549^*luc*^ cell lines stably expressing pri-miRNA21 and Flag-TARBP2-WT or -K52R (**a**, named as miR21-WT or miR21-K52R, respectively), or pri-miR130b and Flag-TARBP2-WT or -K52R (**b**, named as miR130b-WT and miR130b-K52R, respectively) were generated by the lentiviral system. Cell lysates were used for IP with anti-Flag antibody and then detected with anti-Ago2 antibody. Cell lysates were used for immunoblotting with anti-Ago2, -GAPDH, -Flag and -PTEN (**a**) or -ZEB1 (**b**) antibodies. For full scans of western blots, see Supplementary Fig. 8. (**c**) The SUMO-site mutation K52R of TARBP2 dominant-negatively abolishes inhibition of cell migration mediated by miR130b-ZEB1. Cell motilities of stable A549^*luc*^ cell lines including Control, miR130b-con, miR130b-WT and miR130b-K52R were analysed by a wound-healing assay with μ-Dish. (**d**) RIP assay was performed with 293T cells transfected with pri-miR21, myc-Ago2 and Flag-TARBP2-WT or -K52R. Thirty-six hours after transfection, cells were lysed for IP with anti-myc antibody to pull down RNA. Ago2-bound mature miR21 was extracted and analysed by real-time quantitative PCR. (**e**–**g**) SUMOylation of TARBP2 influences the efficiency of miRNA or siRNA mimic duplexes via the formation of the functional RISC. TARBP2-WT or -K52R with/without Ago2, together with miR21 (**e**), miR130b (**f**), mimic duplexes or siPTEN (siRNA for PTEN; **g**) were co-transfected into HeLa cells, and the expression levels of the corresponding targets PTEN, ZEB1 and PTEN were determined. Immunoblotting was performed with anti-PTEN, anti-ZEB1, anti-Myc, anti-Flag and anti-GAPDH antibodies. (**h**) A model for SUMOylation of TARBP2 controls the efficiency of RNA-induced gene silencing by increasing its interaction with Ago2 and precursor miRNAs/siRNAs. ‘S'—SUMOylation; ‘P'—Phosphorylation.

Since above data have confirmed that SUMOylation of TARBP2 promotes its binding with pre-miRNA (Fig. 6c–e) and also stabilizes Ago2 by increasing its binding with Ago2 (Fig. 7), which both probably influence the formation of the functional RISC, we wanted to figure out whether SUMOylation of TARBP2 is directly connected with the efficiency of RISC. Therefore, we performed a dual luciferase report assay to investigate whether SUMOylation of TARBP2 affects the gene-silencing efficiency. We transfected miR21 mimics, sicheck-miR21, Flag-tagged TARBP2, with or without SUMO1+Ubc9 into 293T cells, and then measured the luciferase activities. The relative repression fold of miR21 revealed that SUMOylation of TARBP2 increased the binding of miR21 with its binding sites (Supplementary Fig. 7a). Furthermore, miR21 mimics and sicheck-miR21 with TARBP2-WT or -K52R were transfected into 293T cells for 36 h and then the luciferase activities were measured. The relative repression fold of miR21 showed that TARBP2-WT repressed miR21 binding with its binding sites while TARBP2-K52R abolished this inhibition (Supplementary Fig. 7b). Next, A549^*luc*^ cell lines stably expressing pri-miRNA21 or pri-miR130b were first generated (Supplementary Fig. 7c–d), and further these cell lines were introduced with Flag-TARBP2-WT (named as miR21-WT or miR130b-WT) or Flag-TARBP2-K52R (miR21-K52R or miR130b-K52R). In the above cell lines, mature miR21 or miR130b still did not have significant changes yielded by TARBP2-WT or -K52R by analysis of quantitative real-time PCR. Since PTEN and ZEB1 are reported as the main targets of miR21 (ref. 43 and miR130b (ref. 44), respectively, we chose PTEN and ZEB1 to monitor the efficiency of miRNA-induced gene silencing. Immunoblotting analysis for Input showed that TARBP2-WT improved both miR21 and miR130b, further reducing the protein levels of PTEN (Fig. 8a) and ZEB1 (Fig. 8b), respectively, whereas TARBP2-K52R blocked decreases in the levels of these two proteins by dominant-negatively impeding miRNA-induced gene silencing. These differential effects between TARBP2-WT and TARBP2-K52R with either miR21 or miR130b were supposed to be resulting from the formation of the functional RISC with recruitment of Ago2, the catalytic enzyme of RISC essential for target gene silencing^45^. Indeed, we observed that, as a protein of RISC, the wild-type TARBP2 recruited much more Ago2 compared with the mutant TARBP2-K52R in both miR21 and miR130b systems (Fig. 8a,b, IP panels). Moreover, to assess and validate the effectiveness of RISC by TARBP2 SUMOylation, we also tested phenotypes of miR130b repressing the target ZEB1, which is a core epithelial-to-mesenchymal transition inducing transcription factor and can promote cell migration^46^, with a wound-healing assay for cell migration of stable A549^*luc*^ cell lines stably co-expressing pri-miR130b and TARBP2-WT or -K52R (the same used in Fig. 8b). Consistently with the protein levels of ZEB1 (Fig. 8b), inhibition of cell migration by co-expressing pri-miR130b and TARBP2-WT was more significant than that of cells expressing only pri-miR130b, whereas cells co-expressing pri-miR130b and TARBP2-K52R appeared to have a dominant-negative effect on the suppression of cell migration (Fig. 8c). To get the direct evidence for SUMOyaltion of TARBP2-promoting RISC formation, the RIP assay was performed with 293T cells transfected with pri-miR21, myc-Ago2 and Flag-TARBP2-WT or -K52R. The result of qRT–PCR after IP with anti-myc antibody showed that more mature miR21s were loaded into Ago2 in those co-transfected with TARBP2-WT but not in those co-transfected with TARBP2-K52R (Fig. 8d). Taken together, these data reveal that SUMOylation of TARBP2 recruits Ago2, facilitating the efficiency miRNA-inducing gene silencing.

Since SUMOylation of TARBP2 enhanced RISC-recruiting pre-miRNAs that are stem–loop structure of somehow ‘double-stranded' RNA, we wondered whether TARBP2 SUMOylation also influences the efficiency of miRNA mimic duplexes or siRNA (oligo duplexes) via the formation of the functional RISC. Therefore, we transfected miR21 mimic duplexes and TARBP2-WT or -K52R with or without Ago2 into HeLa cells, and determined the expression level of PTEN targeted by miR21. The expression level of PTEN was downregulated with only miR21 mimics to ∼57–60% (Fig. 8e, lane 2) of the control level in the miR-control mimics (NC). As expected, the co-transfection of miR21 mimics with Ago2 or TARBP2-WT further reduced the expression levels of PTEN to 31% (Fig. 8e, lane 5) or 35% (lane 3), respectively. However, the co-transfection of miR21 mimics with TARBP2-K52R did not reduce more and kept ∼60% (lane 4), indicating that TARBP2-K52R lost the function in the regulation of miRNA-mediated gene silencing. More importantly, the co-transfection of miR21 mimics and Ago2 together with TARBP2-WT maximally reduced the expression levels of PTEN to 22% (lane 6), whereas that with TARBP2-K52R just reduced to 51% (lane 7). Furthermore, we repeated this experiment with miR130b mimic duplexes and measured the expression levels of its target ZEB1, and got the exactly similar pattern of results (Fig. 8f). In addition, the specific siRNA for PTEN was also employed to repeat this same experiment. As expectedly, the similar results as miR21 mimic duplexes (Fig. 8e) for targeting PTEN were observed (Fig. 8g). These data strongly suggest that SUMOylation of TARBP2 is required for its orchestration with AGO2, promoting the efficiency of miRNA- or siRNA-induced gene silencing by recruiting small RNA duplexes.

## Discussion

Small RNA-induced gene silencing is one of the basic molecular mechanisms for the regulation of gene expression and an elaborated complex biological process. However, the underlying mechanisms how the efficiency of miRNA/siRNA-induced gene silencing is fine-tuned represent a critical issue unresolved in the field of noncoding RNA research. Mammalian mature miRNAs are basically generated by a two-step processing. First, pri-miRNAs are cleaved by a microprocessor complex minimally composed of Drosha/DGCR8 to produce ∼70-nt-long stem–loop structural pre-miRNAs in the cell nucleus. Second, pre-miRNAs are exported to the cytoplasm by RanGTP/Exportin5 (refs 4, 6, 47), and then are recognized and cleaved by the Dicer/TARBP2 complex into the duplex miRNAs^7^,^48^, which are sequentially embedded into Ago2, a core component of the RISC complex^5^,^49^,^50^. There is increasing evidence that RISC assembly contains two key steps including the duplex miRNA loading into RISC and the miRNA duplex unwinding for gene silencing. In the latter, one strand of the miRNA duplex is selected by Ago2 as the guide strand, which is incorporated into Ago2 to form the functional centre of the RISC complex^8^,^51^,^52^, while another is the passenger strand to be degraded.

TARBP2 can assemble with Dicer and Ago2 to form RLC, which is essential for the efficient transfer of nascent siRNAs and miRNAs from Dicer to Ago2 (refs 7, 8, 52, 53). In this study, we provided evidence that TARBP2 was SUMOylated at K52 (Figs 1 and 2). External signals first activated phosphorylation of TARBP2 via the MAPK/ERK signalling pathway, and subsequently upregulated TARBP2 SUMOylation (Fig. 3), which stabilized TARBP2 itself by inhibition of the K48-linked ubiquitination of TARBP2, thereby preventing the degradation from the ubiquitin proteasome pathway (Fig. 4). Although SUMOylation of TARBP2 appeared not to have an influence on the miRNA production (Fig. 6a,b and Supplementary Data 1), it regulated miRNA/siRNA silencing efficiency. Mechanically, SUMOylated TARBP2 together with Dicer recruited more Ago2 to constitute RLC through the direct interaction of the SUMO1 conjugated to TARBP2 with the SIMs of Ago2 (Fig. 7a–e). Simultaneously, SUMOylation of TARBP2 promoted more pre-miRNAs or siRNAs loading into the RLC (Fig. 6c–e and Supplementary Fig. 5). These brought about that Ago2 was more stabilized (Fig. 7f–g), and miRNAs/siRNAs bound by TARBP2/DICER were efficiently transferred to Ago2. Thus, the miRNA guide strands or siRNAs were highly loaded into Ago2 to form the functional centre of the RISC complex, which significantly improved the efficiency of RISC to silence specific mRNAs by degradation or translational inhibition (Fig. 8a–g). To our knowledge, this work is the first one to prove that TARBP2 can be modified by SUMO1 and this modification regulates the efficiency of miRNA/siRNA, as summarized in Fig. 8h.

Two Dicers, Dcr-1 and Dcr-2 in *Drosophila*, have clearly different functions^54^. Dcr-1 is critical for miRNA processing, while Dcr-2 acts as a major siRNA-processing enzyme. R2D2 in *D. melanogaster* can interact with Dcr-2 as a bridge between initiation and effective steps of RNA interference (RNAi)^55^, and R2D2 also directly binds to double-strand siRNA with Dcr-2 and helps Ago2 in sensing the guide strand of siRNA, ensuring that the authentic siRNA enters the RNAi pathway^56^. However, in mammals, there is only one RNAase type III Dicer that can process both siRNAs and miRNAs^57^. It is widely known that TARBP2 is an important partner of Dicer, and binds to pre-miRNAs and recruits Dicer for cleavage^7^,^11^,^19^,^52^,^58^,^59^. However, interestingly, TARBP2 is not a homologue of R2D2 but of LOQS^9^,^60^. Our data proved that SUMOylation of TARBP2 promoted its binding with pre-miRNAs, but had little influence on the expression patterns of mature miRNAs (Fig. 6a and Supplementary Data 1). This was because the SUMO-site mutation K52R of TARBP2 did not affect the interaction between TARBP2 and Dicer (Supplementary Fig. 6d), and Dicer is in charge of processing of most miRNAs; thus, the limited changes in production of mature miRNAs was not unexpected.

SUMOylation of TARBP2 influenced its interaction with Ago2 (Fig. 7a), a core component of RISC^7^,^61^,^62^, other than PKR, KSRP and PACT (Supplementary Fig. 6a–c). The mutation K52R of TARBP2 impaired its binding with either exogenous or endogenous Ago2 (Fig. 7a–c), whereas TARBP2 SUMOylation increased the interaction between TARBP2 and Ago2 via the binding of SUMO1 conjugated to TARBP2 with SIMs of Ago2 (Fig. 7d,e). The concept of SIMs is a widely accepted theory to explain this phenomenon caused by SUMOylation^39^,^63^. There are at least 18 SIMs in Ago2 according to the prediction made by using the GPS-SUMO software^42^. In this study we chose two of them, SIM1 (162–166 aa) and SIM2 (519–523 aa), as potential sites and verified that the interaction between TARBP2 and Ago2 could be regulated by SUMOylation. The structure modelling of AGO2 revealed that SIM1 was located between N- and L1 domains, where it was partially buried in the hydrophobic core of Ago2; thus, it may have some opportunities for SUMO1 conjugated on TARBP2 to bind with Ago2. Moreover, the crystal structure of Ago2 has revealed that MID and PIWI domains bind to guide strands of small RNAs, and specially Y^529^ can form a hydrogen bond with the 5′-base of RNA. Besides Y^529^ in the MID domain, other three amino-acid residues, K^533^/N^545^/K^566^, are also important for binding with small RNAs^64^,^65^. Since these residues are close to the Ago2 SIM2 (aa 519–523), SUMOylation of TARBP2 may also help in Ago2 binding and selecting guide strand of miRNAs more efficiently. Thus, SUMOylated TARBP2, probably together with Dicer, recruited more Ago2 to provide a platform RLC, comprising the proteins Ago2, Dicer and TARBP2 (ref. 53), for targeted gene silencing by RNAi. In addition, Ago2 was stabilized by SUMOylation of TARBP2 in cells (Fig. 7f,g).

As TARBP2 binds to a stem-like structure of double-stranded RNA, which can be extended to ≈22-nt RNA duplexes (immature miRNAs or siRNAs) yielded from the cleavage of pre-miRNAs or double-stranded RNAs by Dicer. The immature miRNA duplexes lie in the state between pre-miRNA and mature miRNA^66^,^67^. At this stage, miRNA/siRNA duplexes can be assembled with RLC^68^, whose core element Ago2 subsequentially functions to sense and select the guide strand of a small RNA duplex. As both dsRBD of TARBP2 can bind to immature miRNAs independently^66^ and the SUMO-site K52 of TARBP2 is exactly located in dsRBD1, indicating that in humans SUMOylated TARBP2 might play a similar role as R2D2^56^ in loading siRNA/miRNA duplex into the Ago2-containing complex RISC, which triggers the siRNA/miRNA-induced RNAi pathway. Although SUMOylation of TARBP2 was indispensable for TARBP2 binding with pre-miRNAs (Fig. 6c–e), we also found that TARBP2 SUMOylation significantly influenced the efficiency of miRNA mimic duplexes or siRNA via the formation of the functional RISC (Fig. 8d–f), demonstrating that SUMOylation of TARBP2 was required for its orchestration with AGO2 recruiting small RNA duplexes, thus promoting the efficiency of miRNA- or siRNA-induced gene silencing.

## Methods

### Antibodies and reagents

Antibodies against TARBP2 (15753-1-AP) and His-tag (66005-1-Ig) were obtained from ProteinTech Group (Wuhan, China). Antibody Anti-GST (CW0084) was purchased from CWbioTech (Shanghai, China). Antibodies against Ago2 (C34C6; #2897), ZEB1 (#3396), PTEN (#9188), Dicer (#3363), Phospho-p44/42-Erk1/2 (#4370), p44/42-Erk1/2 (137F5; #4695), Myc (#2276), GFP (#2956) and β-Actin (13E5) were from Cell Signaling Technology. Anti-Flag M2 (F1804) and anti-HA (MMS-101 P) mouse antibodies were from Sigma. Antibodies against GAPDH (#ab37168), SUMO1 (Y299; #ab32058) Senp1 (EPR3844; #ab108981) were from Abcam. Protein G Plus/Protein A agarose suspension (#IP05) was purchased from Calbiochem. Ni^2+^-NTA agarose beads were obtained from Qiagen (Hilden, Germany). Glutathione Sepharose 4B (#17-0756-01) was from GE Healthcare Life Sciences (NJ, USA). CHX (#C7698), U0126 (#U120), hydrogen peroxide solution (H_2_O_2_, #H1009), polybrene (hexadimethrine bromide, # H9268) and puromycin (#P8833) were from Sigma. MiR21 mimics, miR130b mimics and siPTEN were obtained from GenePharma (Shanghai, China).

### Plasmids

The pLPC-TARBP2 plasmid was kindly provided by Dr Sonia A. Melo and then subcloned into the vectors pCMV-Tag2b and pCMV-myc. The Flag-TARBP2 was cloned into the lentiviral vector (System Biosciences, Mountain View, CA, USA) carrying *EGFP* and *Puromycin* genes^28^,^46^. The TARBP2 cDNA was cloned into the prokaryotic expression vector pGEX4T1. The shRNA sequence targeting human TARBP2 3′-UTR (TARBP2sh) was obtained from Sigma ‘Mission shRNA' online: 5′-TCATGGATGTGCACCCTTTG-3′ at 1,604 site. The shRNA was cloned into pLKO.1 vector or pGreenPuro shRNA (System Biosciences). The oligo sequences of other shRNAs including Senp1sh1, Senp1sh2 and SUMO1sh are listed in Supplementary Table 2.

### Cell cultures

HEK293T, 293FT, A549 and HeLa cell lines were cultured in DMEM (obtained from Hyclone) containing 10% fetal calf serum (Biowest, Kansas, MO, USA), penicillin and streptomycin (Invitrogen, CA, USA). Human prostate cancer cell line M12 was cultured in RPMI 1640 (Hyclone) plus 5% FBS, 2.5 μg ml^−1^ Fungizone (Sagon, Shanghai, China), 10 ng ml^−1^ epidermal growth factor (Roche), 0.2 μM dexamethasone (Sigma), 5 μg ml^−1^ insulin (Sigma), 5 μg ml^−1^ transferrin (Sigma), 5 ng ml^−1^ sodium selenite (Sigma) and 50 μg ml^−1^ gentamycin (Sagon). All cells were cultured at 37 °C in a 5% CO_2_-humidified incubator. A549^*luc*^ and M12^*luc*^ stably expressing a firefly luciferase were used for living imaging. Cell transfection was performed using Lipofectamine 2000 (Invitrogen).

### SUMOylation assays

Three methods were used to determine TARBP2 SUMOylation. TARBP2 SUMOylation *in vivo* was analysed in 293T or HeLa cells with the method of his-tagged SUMO1 binding to Ni^2+^-NTA beads^27^,^28^,^31^. *In vitro* SUMOylation assay in *Escherichia coli* system with pE1E2S1 was performed as previously described^30^,^31^. The method of co- IP/western blotting was also used to detect SUMOylation of TARBP2. Briefly, GFP-SUMO1, HA-Ubc9 and Flag-TARBP2 were transfected into 293T cells. Forty-eight hours after transfection, cells were lysed in NEM-RIPA buffer (50 mM Tris-HCl pH 7.4, 150 mM NaCl, 1% NP-40, 20 mM N-ethylmaleimide and a complete protease inhibitor cocktail)^27^ for IP. Cell lysates (1 mg) were incubated with 30 μl of 50% slurry of protein A/G agarose beads and 2 μl of anti-Flag antibody at 4 °C overnight, followed by immunoblotting with anti-GFP and anti-Flag (dilution 1:1,000).

### GST protein pull-down assays

This method has been previously described^46^. Briefly, *E. coli* BL21 expressing GEX4T1-TARBP2-WT or -K52R was lysed with B-PER Protein Extraction Reagent (#78248, Thermo Fisher, USA). Lysates were incubated with Glutathione sepharose 4B (GE Healthcare) overnight at 4 °C and washed five times with lysis buffer. Western blot analysis was employed for detecting the interactions between the special proteins and GST-tagged TARBP2.

### Analysis of mature miRNAs and pre-miRNAs with qPCR

Total RNA was extracted with TRIZOL regeant (Invitrogen) following instructions. Quantitative real-time PCR (qRT–PCR) was performed as described^38^. U6 small nuclear RNA and GAPDH were used for normalization of miRNA and pre-miRNA PCRs, respectively.

### RNA IP assay

RNA-binding assay and qRT–PCR were modified from previous study^69^. Briefly, after transfection, cells were lysed with RIP lysis buffer (150 mM NaCl, 50 mM Tris-HCl pH 7.4, 1% NP40, 1 mM dithiothreitol, 100 units ml^−1^ RNase inhibitor (Fermentas), 400 uM VRC (New England BioLabs) and Protease inhibitor cocktail (Roche)). After 30 min of lysis on ice, one-tenth of the lysates was used as input. Other lysates were incubated with protein A/G agarose beads and 2 μl of anti-Flag antibody at 4 °C. After IP, the beads were washed five times with RIP lysis buffer. Total RNAs were extracted withTRIZOL reagent (Invitrogen) following instructions. Purified RNAs were used to perform the reverse transcriptional PCR using the PrimeScript RT-PCR Kit (#RR037A, TAKARA). qRT–PCR was performed with SYBR Green PCR Master Mix (#4309155, Applied Biosystems, USA) to analyse the RNA–protein-binding fold changes. U6 small nuclear RNA and GAPDH levels were used for normalization of miRNA and pre-miRNA PCRs, respectively.

### Migration assay by wound healing

This method for analysis of migration was conducted as described previously^31^. Briefly, 5 × 10^3^ of each of serum-starved A549^*luc*^ stable cell lines were seeded into the μ-Dish (35 mm high, purchased from IBIDI) and cultured overnight for adhering before a clear area was created by removing the Culture-Insert from the μ-Dish. Photos were taken at the indicated time.

### Migration assay by RTCA-DP

The procedure of the xCELLigence RTCA-DP system (Roche) was previously described^31^,^70^. Briefly, 2 × 10^4^ of each of serum-starved A549^*luc*^ stable cell lines were suspended in 100 μl of serum-free medium, and then the cell suspension was added into the pre-equilibrated upper chamber of the CIM plate. The complete medium was added to the bottom-well of the plate, and a FBS-free medium was used as control. The plate was then inserted into the RTCA machine (housed within the incubator) and cell index values were detected every 15 min over the following procedure. The slopes of the curves at indicated time points were calculated using the RTCA software v1.2 (Roche Applied Science).

### Soft agar colony formation assay

The effect of TARBP2-WT and -K52R on cellular transformation and tumorigenesis was assessed using a soft agar colony assay as previously described^28^,^46^. Briefly, this assay was performed in six-well plates containing 2 ml of 0.6% base agar gel (Amresco) with 10% FBS. Cells were seeded at a density of 2 × 10^3^ cells per well in 1.5 ml of 0.35% agar gel with 10% FBS and were layered on the base gel. The photographs of the cell colonies developed in soft agar were taken at an indicated day after crystal violet staining and the number of colonies was counted using ImageJ (NIH, USA). At least three independent experiments were performed in triplicate.

### Xenograft tumour model

The experiment of xenograft tumour model was conducted as previously described^28^. Each of stable A54*9*^*luc*^ cell line expressing the control vector, or Flag-TARBP2-WT, or Flag-TARBP2-K52R (at the final concentration of 2.5 × 10^6^ cells/each) was injected subcutaneously into 5-week-old male BALB/c nude mice (*n*=4) individually. Tumors were assessed by imaging isofluorane-anaesthetized mice with the IVIS system (Xenogen). Images were obtained 10 min after intraperitoneal injection of 1.5 mg (∼75 mg kg^−1^) D-luciferin (Xenogen) in 100 μl of PBS. The light emitted by luciferase-expressing tumour was quantified using Living Image v2.50 (Wavemetrics). Mice were killed after living image to remove tumours. Tumours were weighted and photographed. All animal studies were conducted with the approval and guidance of Shanghai Jiao Tong University Medical Animal Ethics Committees.

### Statistical analysis

All data are presented as means±s.d. for western blotting (Fig. 8d–f), or means± s.e.m. for qPCR, RTCA migration, mouse xenograft model and soft agar colony assay. Statistical calculations were performed with Microsoft Excel analysis tools. Differences between individual groups are analysed using the *t*-test (two-tailed and unpaired). A *P* value of <0.05 (*), <0.01 (**) or<0.001 (***) is considered significant.

## Additional information

**How to cite this article:** Chen, C. *et al.* SUMOylation of TARBP2 regulates miRNA/siRNA efficiency. *Nat. Commun.* 6:8899 doi: 10.1038/ncomms9899 (2015).

## Supplementary Material

Supplementary Information Supplementary Figures 1-8 and Supplementary Tables 1-2

Supplementary Data 1 All miRNA expression profile of A549luc cell lines stably expressing Flag-tagged TARBP2-WT or TARBP2-K52R by High-throughput sequencing
