# Supplementary material for: SUMOylation of TARBP2 regulates miRNA/siRNA efficiency
Source: Nat Commun. 2015 Nov 19;6:8899. doi: 10.1038/ncomms9899 (PMC4673853; doi:10.1038/ncomms9899)
Supplement: Supplementary Information — Supplementary Figures 1-8 and Supplementary Tables 1-2 [file ncomms9899-s1.pdf]

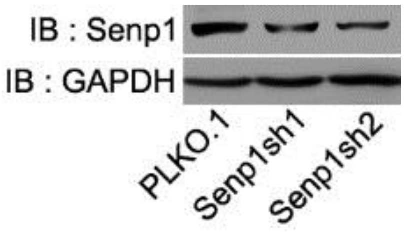

Supplementary Figure 1. Generation of stable Senp1 knock-down 293T cell lines. HEK293T were infected by the lentiviral system carrying shRNA1 or shRNA2 for knock-down of Senp1 (Senp1sh1 or Senp1sh2). Knock-down efficiency of Senp1 was determined by Western blotting for efficiency. The stable cell line with Senp1sh2 showing lower expression of Senp1 was selected for the following experiments. This is related to Fig. 1d.

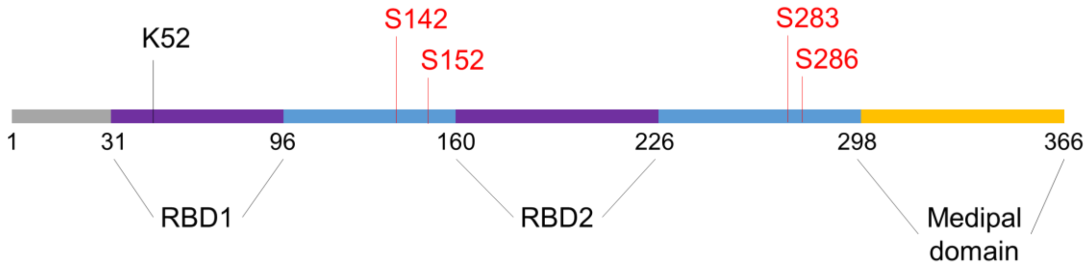

Supplementary Figure 2. Phosphorylation and SUMOylation sites of TARBP2. Four phosphorylation sites including S^{142}, S^{152}, S^{283} and S^{286} , and one SUMO-site K^{52} are shown. This is related to Fig. 3a.

a

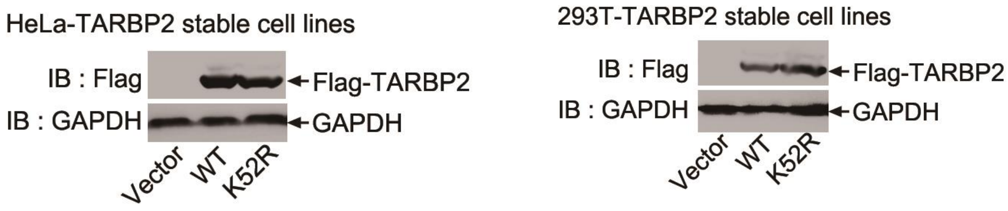

b

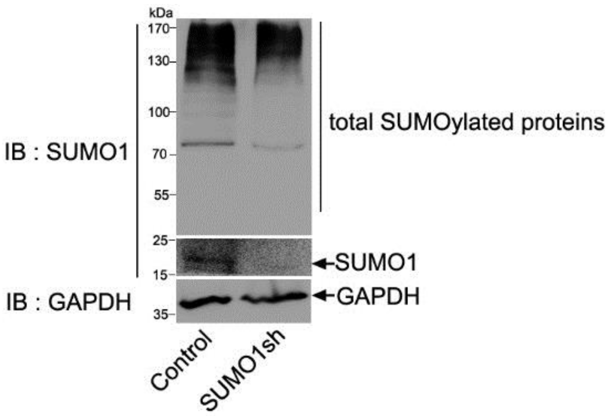

Supplementary Figure 3. (a) HeLa and 293T cell lines stably expressing Flag-tagged TARBP2-WT or -K^{52}R were generated with the lentiviral system. Ectopic expression of Flag-TARBP2 in stable HeLa and 293T cell lines were detected by immunoblotting with anti-Flag antibody. This is related to Fig. 4d. **(b) SUMO1 knock-down 293T stable cell lines**. SUMO1 was stably knocked down in 293T cells (SUMO1sh). The expression levels of SUMO1 and total SUMO1-modified proteins in control and SUMO1sh cell lines were determined by immunoblotting with anti-SUMO1 antibody. This is related to Fig. 4e.

a

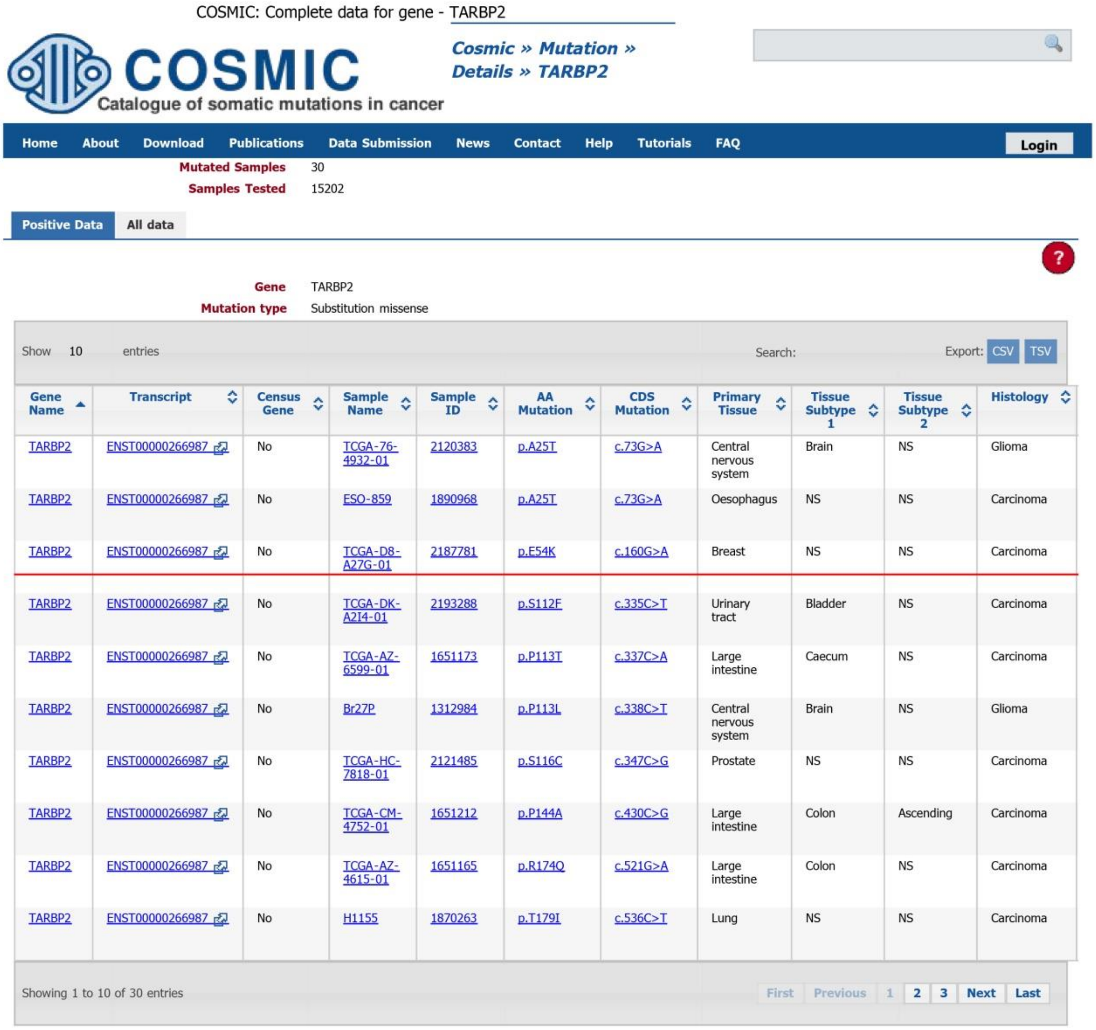
COSMIC: Complete data for gene - TARBP2

COSMIC
Catalogue of somatic mutations in cancer

Cosmic » Mutation » Details » TARBP2

Home About Download Publications Data Submission News Contact Help Tutorials FAQ Login

Mutated Samples 30
Samples Tested 15202

Positive Data | All data

Gene TARBP2
Mutation type Substitution missense

Show 10 entries Search: Export: CSV TSV

Gene Name	Transcript	Census Gene	Sample Name	Sample ID	AA Mutation	CDS Mutation	Primary Tissue	Tissue Subtype 1	Tissue Subtype 2	Histology
TARBP2	ENST00000266987	No	TCGA-76-4932-01	2120383	p.A25T	c.73G>A	Central nervous system	Brain	NS	Glioma
TARBP2	ENST00000266987	No	ESO-859	1890968	p.A25T	c.73G>A	Oesophagus	NS	NS	Carcinoma
TARBP2	ENST00000266987	No	TCGA-D8-A27G-01	2187781	p.E54K	c.160G>A	Breast	NS	NS	Carcinoma
TARBP2	ENST00000266987	No	TCGA-DK-A2I4-01	2193288	p.S112F	c.335C>T	Urinary tract	Bladder	NS	Carcinoma
TARBP2	ENST00000266987	No	TCGA-AZ-6599-01	1651173	p.P113T	c.337C>A	Large intestine	Caecum	NS	Carcinoma
TARBP2	ENST00000266987	No	Br27P	1312984	p.P113L	c.338C>T	Central nervous system	Brain	NS	Glioma
TARBP2	ENST00000266987	No	TCGA-HC-7818-01	2121485	p.S116C	c.347C>G	Prostate	NS	NS	Carcinoma
TARBP2	ENST00000266987	No	TCGA-CM-4752-01	1651212	p.P144A	c.430C>G	Large intestine	Colon	Ascending	Carcinoma
TARBP2	ENST00000266987	No	TCGA-AZ-4615-01	1651165	p.R174Q	c.521G>A	Large intestine	Colon	NS	Carcinoma
TARBP2	ENST00000266987	No	H1155	1870263	p.T179I	c.536C>T	Lung	NS	NS	Carcinoma

Showing 1 to 10 of 30 entries First Previous 1 2 3 Next Last

b

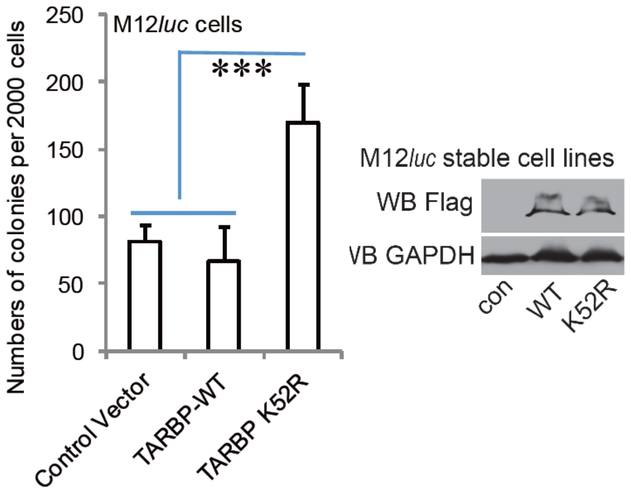

c

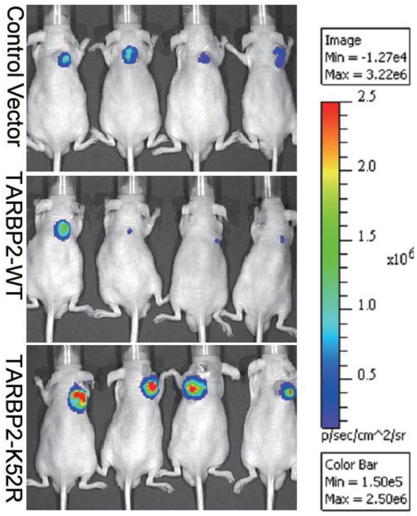

Supplementary Figure 4. (a) Somatic mutations of TARBP2 in patient tissue provided by COSMIC. Red line indicates the ^{54}Glumate-to-Lysine mutation (E54K), which is in the evolutionarily conserved SUMOylation motif ^{51}LKAE^{54} (ΨKXD/E). This is related to Fig. 5a. **(b) SUMOylation of TARBP2 suppresses anchorage-independent growth.** Each of M12^{luc} stable cell line expressing control vector, or Flag-TARBP2-WT, or Flag-TARBP2-K^{52}R (right panels) were seeded at density of 2000 cells/well in 1.5 ml of medium containing 10% FBS with 0.35% soft agarose and layered onto 0.6% solidified agarose. Photographs were taken 12 days after seeding, and the number of colonies was scored. Each value represents the mean ± s.e.m. of three independent experiments with triplicates (left panels). This is related to Fig. 5b. **(c) SUMOylation of TARBP2 suppresses tumor growth *in vivo*.** Each of stable A549^{luc} cell line expressing control vector, or Flag-TARBP2-WT, or Flag-TARBP2-K^{52}R (2.5×10^6 cells/each) was injected subcutaneously into male BALB/c nude mice (n=4) individually. Tumor was assessed by bioluminescent imaging with Xenogen IVIS imaging system 4 weeks before sacrificed. This is related to Fig. 5c.

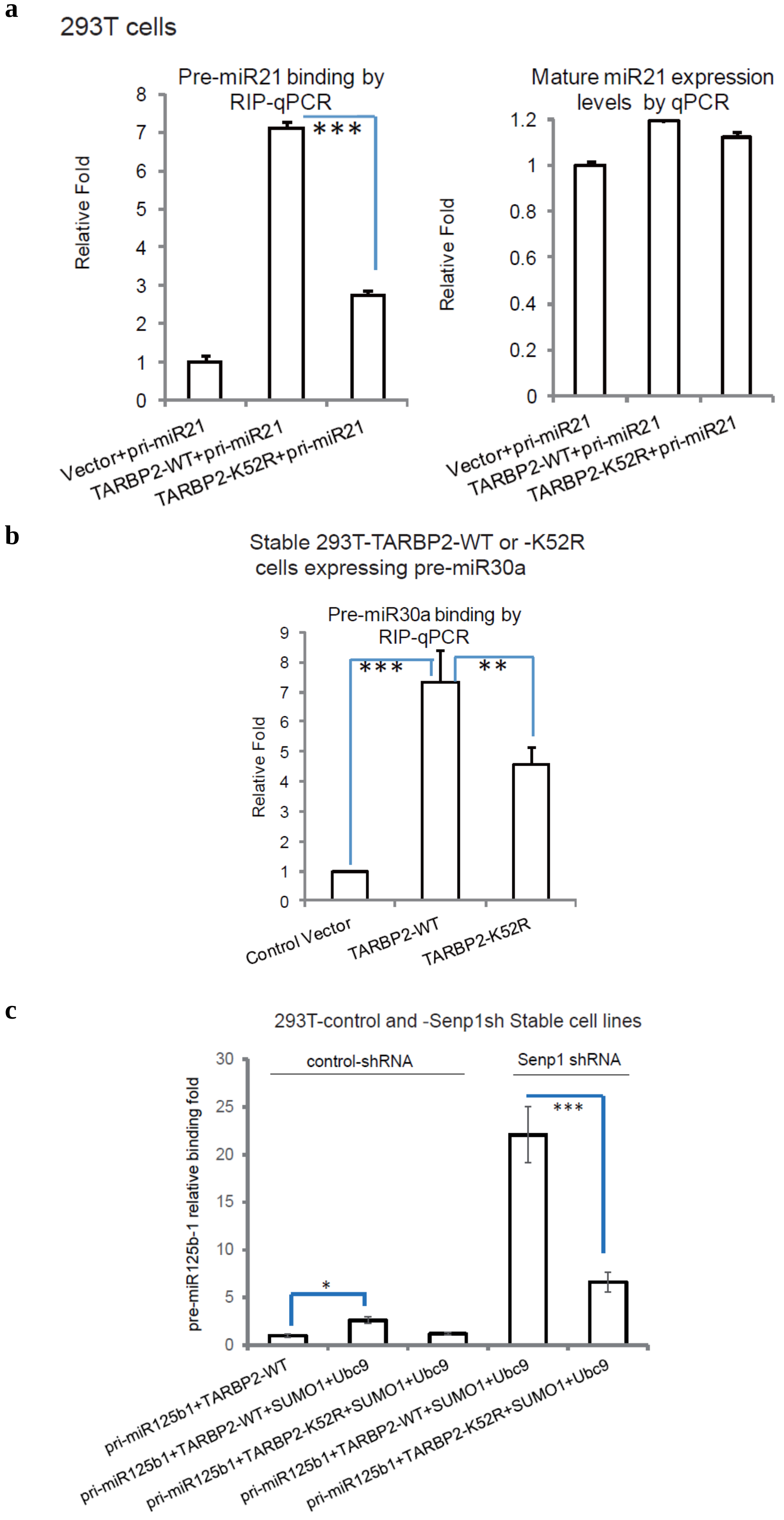

a
293T cells
Pre-miR21 binding by RIP-qPCR
Relative Fold
***
Vector+pri-miR21
TARBP2-WT+pri-miR21
TARBP2-K52R+pri-miR21
Mature miR21 expression levels by qPCR
Relative Fold
Vector+pri-miR21
TARBP2-WT+pri-miR21
TARBP2-K52R+pri-miR21
b
Stable 293T-TARBP2-WT or -K52R cells expressing pre-miR30a
Pre-miR30a binding by RIP-qPCR
Relative Fold
***
**
Control Vector
TARBP2-WT
TARBP2-K52R
c
293T-control and -Senp1sh Stable cell lines
control-shRNA
Senp1 shRNA
pre-miR125b-1 relative binding fold
*
***
pri-miR125b1+TARBP2-WT
pri-miR125b1+TARBP2-WT+SUMO1+Ubc9
pri-miR125b1+TARBP2-K52R+SUMO1+Ubc9
pri-miR125b1+TARBP2-WT+SUMO1+Ubc9
pri-miR125b1+TARBP2-K52R+SUMO1+Ubc9

Supplementary Figure 5. SUMOylation promotes TARBP2 binding with Pre-miRNAs. RNA-binding assay in HEK293T cells. **(a)** Pri-miR21 with TARBP2-WT or -K^{52}R were transiently transfected into 293T cells. 36 h after transfection, cells were lysed for immunoprecipitation with anti-Flag antibody to pulldown RNA. Bound RNA was extracted and analyzed by quantitative PCR for pre-miR21. Left panel: the relative fold of pre-miR21 binding with Flag-TARBP2-WT or -K^{52}R. Right panel: as a control, the expression levels of mature miR21 were analyzed with total RNAs by quantitative PCR. This is related to Fig. 6c. **(b)** Stable 293T cell lines expressing control vector, Flag-TARBP2-WT or -K52R were transiently transfected with pre-miR30a. The RIP assay was performed as (A), and the relative fold of pre-miR21 binding with Flag-TARBP2-WT or -K^{52}R was shown. This is related to Fig. 6d. **(c)** Stable control or Senp1 shRNA knock-down 293T cell lines were transfected with indicated plasmids. 36 h after transfection, cells were lysed and the RNA-binding assay was performed. The relative fold of pre-miR125b1 binding with TARBP2 was shown by analysis of quantitative PCR. This is related to Fig. 6e.

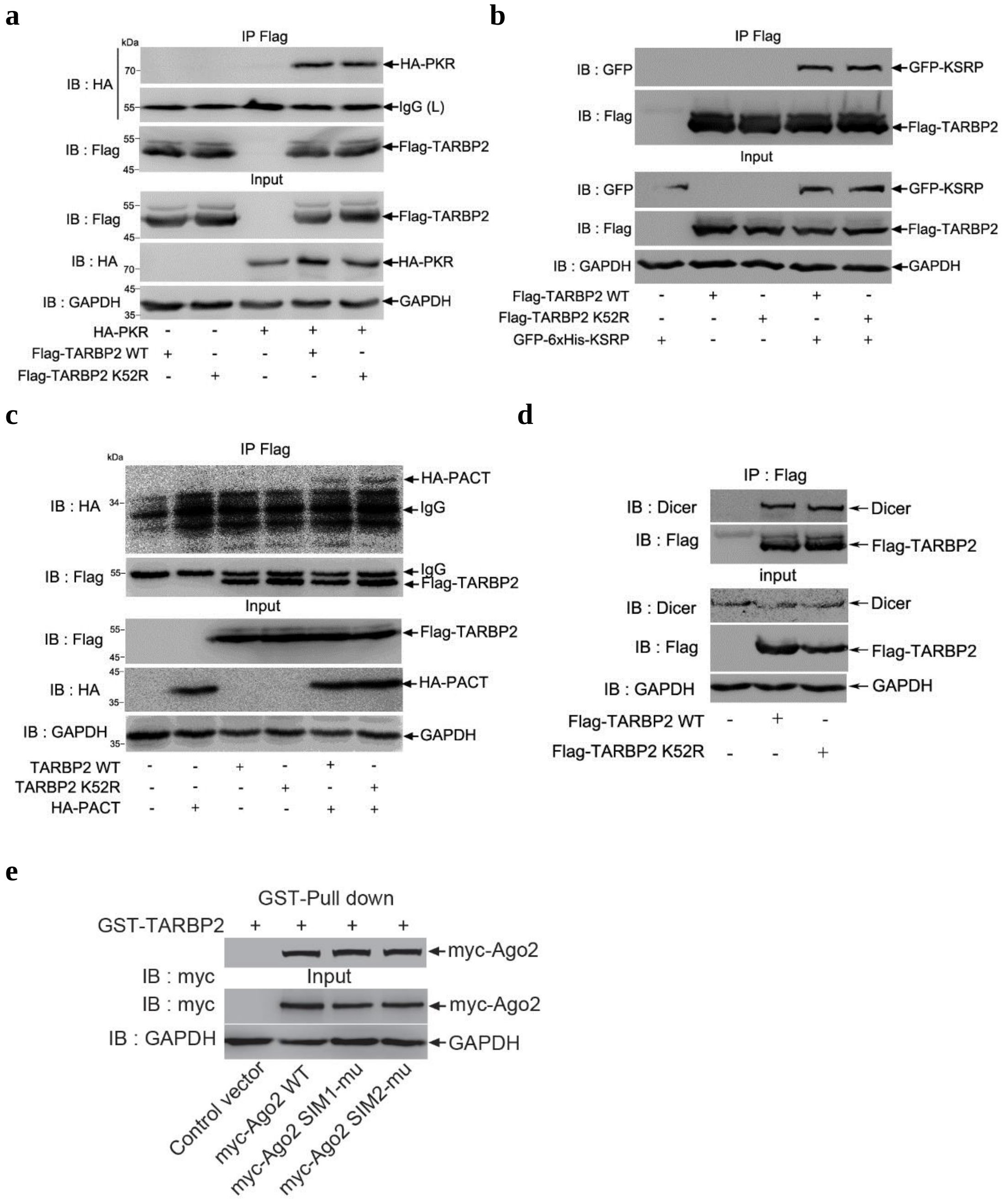

Supplementary Figure 6. **(a-d)** The SUMO-site mutation $K^{52}R$ of TARBP2 does not affect the interaction of TARBP2 with PKR, KSRP, PACT and DICER. Flag-TARBP2-WT or -$K^{52}R$ together with/without HA-PKR **(a)** or GFP-KSRP **(b)** or HA-PACT **(c)**, respectively, were transfected into 293T cells. Cell were lysed for immunoprecipitation with anti-Flag antibody, and then immunoblotted with anti-HA for PKA **(a)**, anti-GFP for KSRP **(b)**, anti-HA for PACT **(c)** and anti-DICER for endogenous Dicer **(d)**, respectively. All cell lysates as Input were detected by immunoblotting. These are related to Fig. 7a. **(e)** The binding of GST-TARBP2

expressed (without pE1E2S1) with either Ago2-WT or two SIM-mutants was comparable. pGEX4T1-TARBP2 without pE1E2S1 was transfected into *E.coli* BL21 (DE3). GST-TARBP2 protein was purified for pull-down of the same amount of each lysate from 293T cells transfected control vector, myc-Ago2 WT or SIM1-mu or SIM2-mu, followed by immunoblotting with anti-Myc antibody. Cell lysates were also used as an Input. This is related to Fig. 7e.

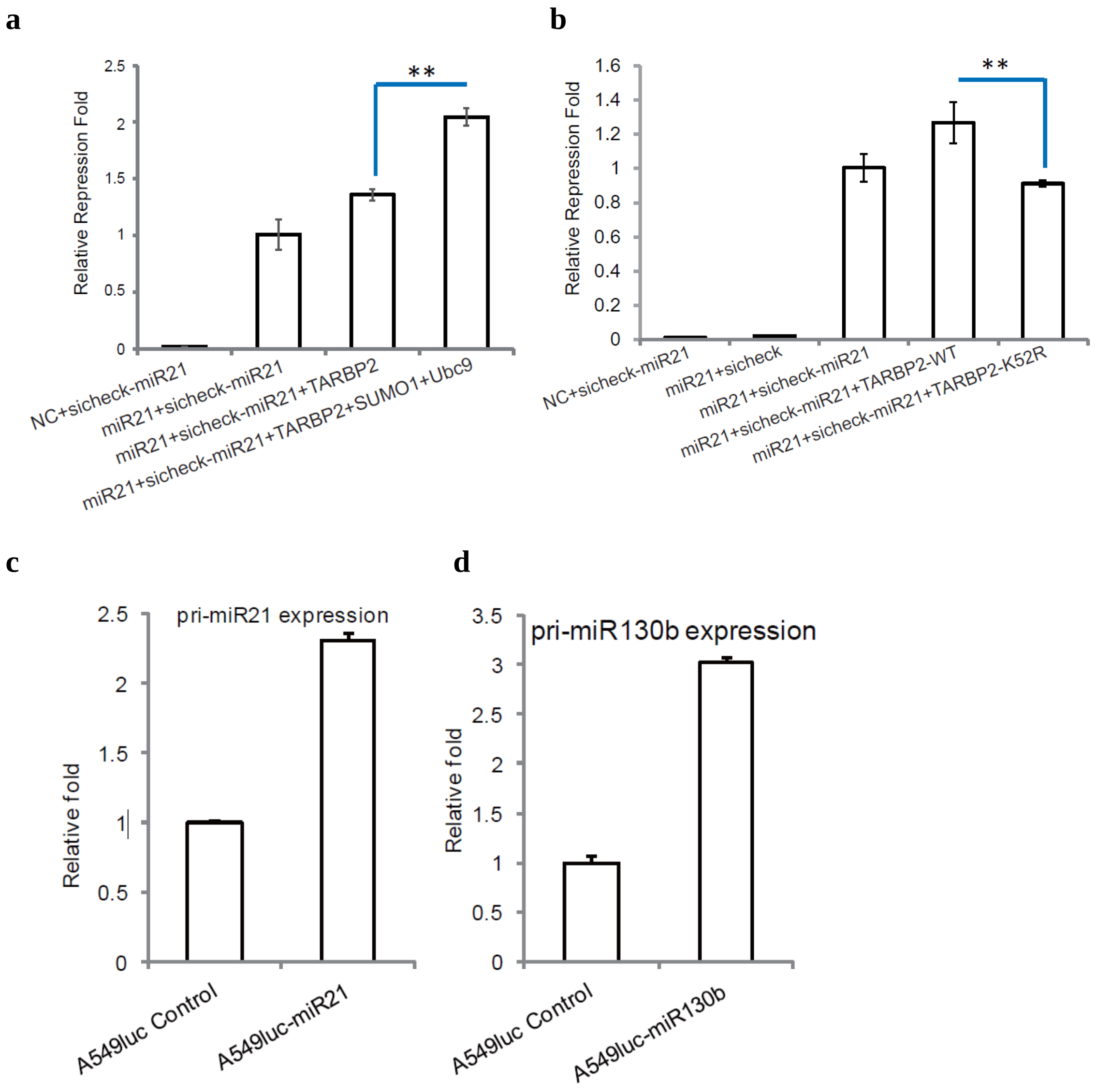

Supplementary Figure 7. (a-b) Dual luciferase assays were performed with 293T transfected with indicated vectors. Firefly and renilla luciferase activities were assayed with the dual luciferase assay system (Promega). Firefly luciferase activity was normalized to renilla luciferase activity following the manufacturer. Three independent experiments were performed in triplicate. **(c)** The expression of pri-miR21 in the stable A549^{luc} cell line stably expressing pri-miRNA21 (A549^{luc}-miR21) was examined by quantitative PCR. This is related to Fig. 8A. **(d)** The expression of pri-miR130b in the stable A549^{luc} cell line stably expressing pri-miR130b (A549^{luc}-miR130b) was examined by quantitative PCR. This is related to Fig. 8b.

Supplementary Figure 8. Full scans of Western blots in this study

Fig. 1b

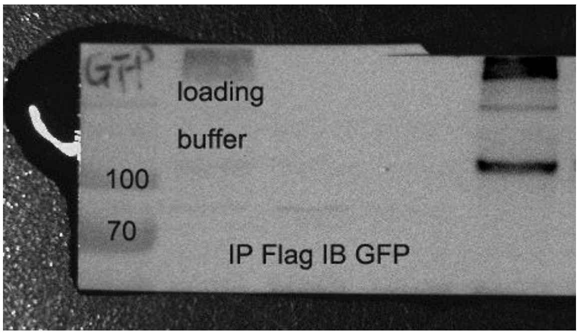

Fig.1d

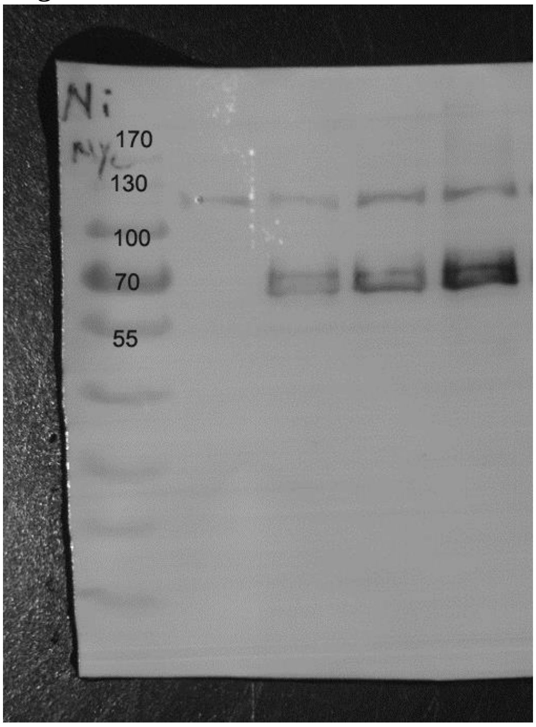

Fig. 1e

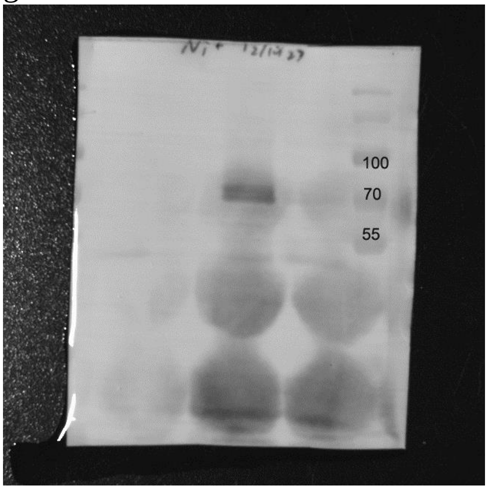

Fig.1f

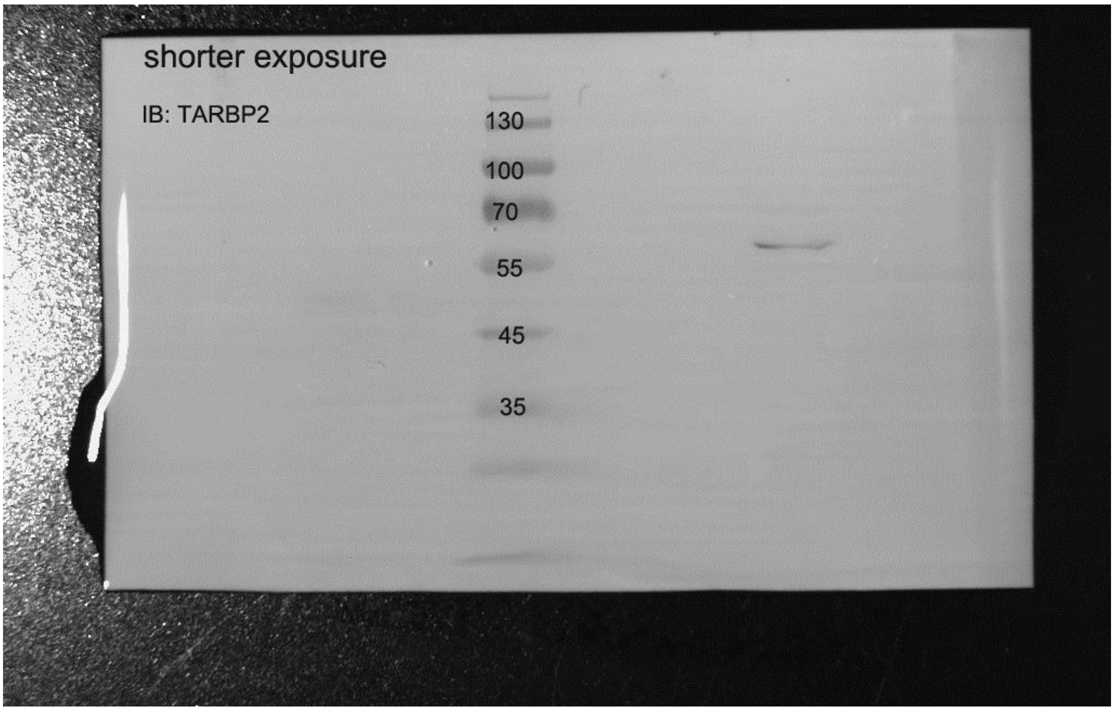

Fig. 2a

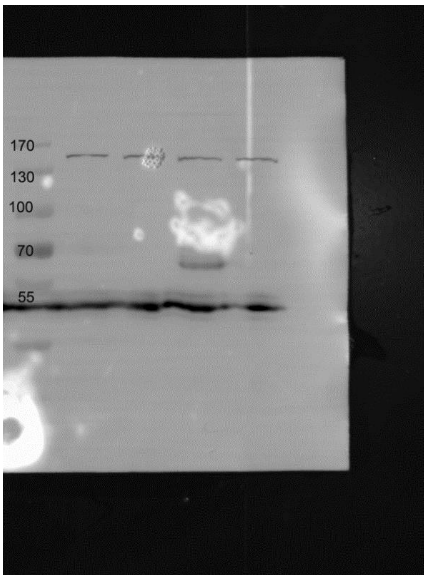

Fig. 2c

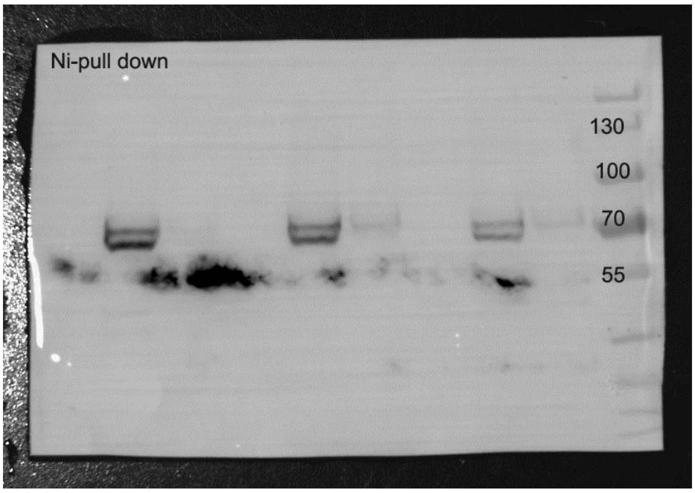

Fig. 2b

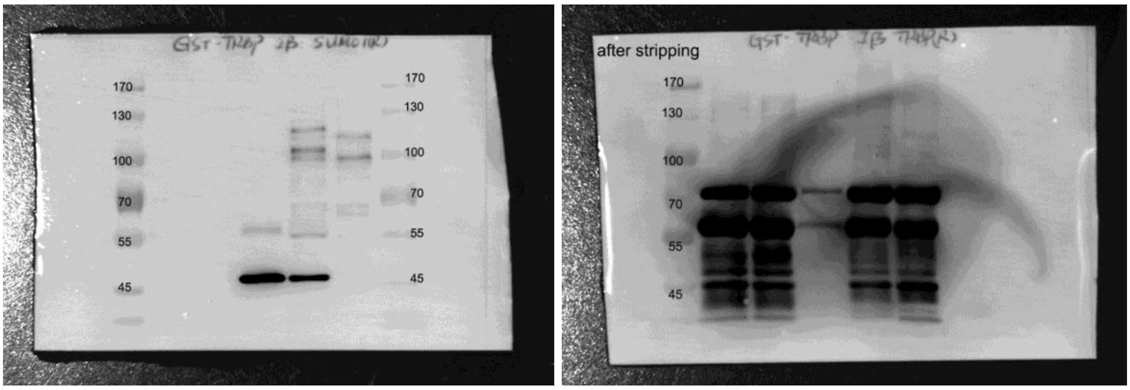

Fig. 2d

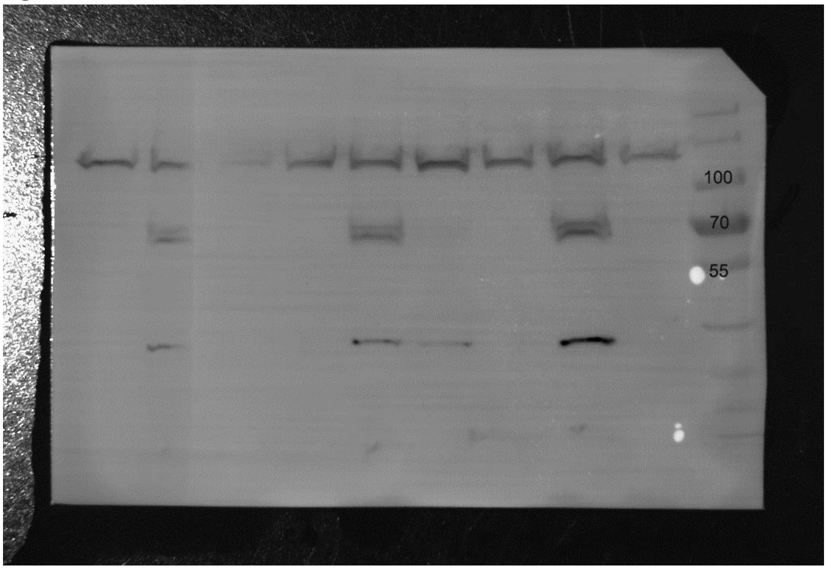

Fig. 3a

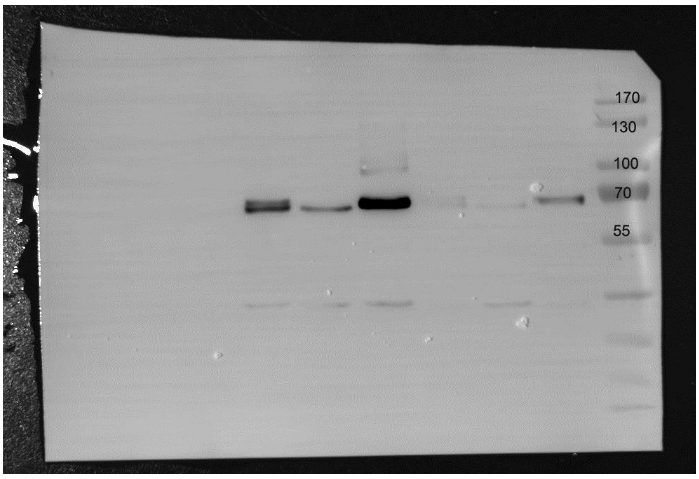

Fig. 3b

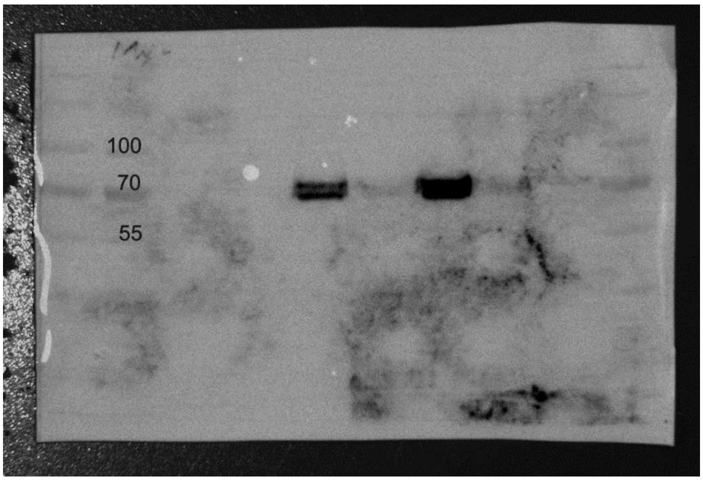

Fig. 3c

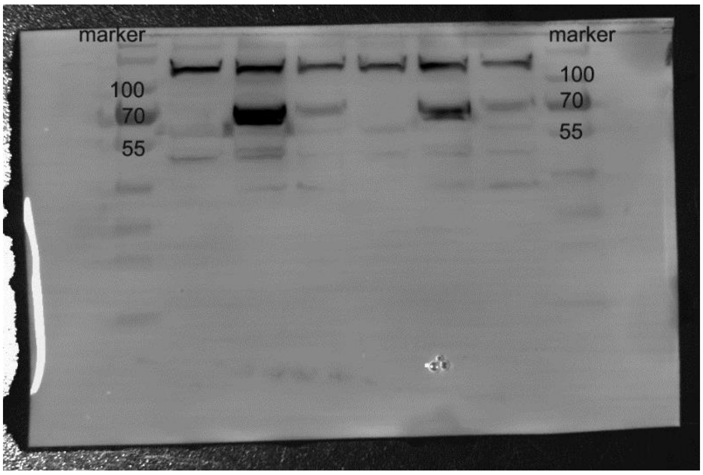

Fig. 4a

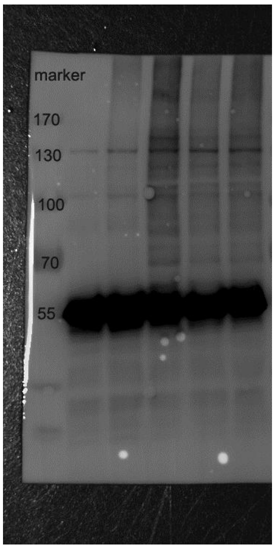

Fig.4b

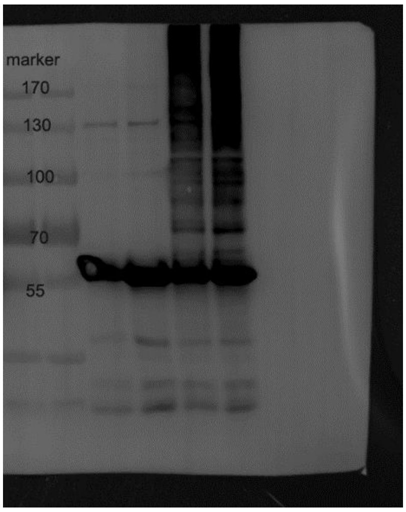

Fig. 4d

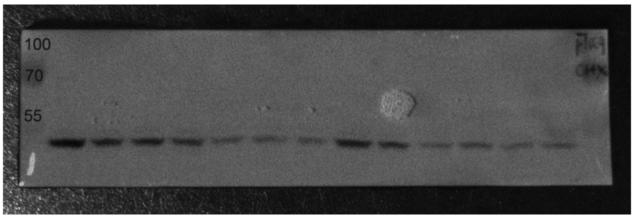

Fig. 5a

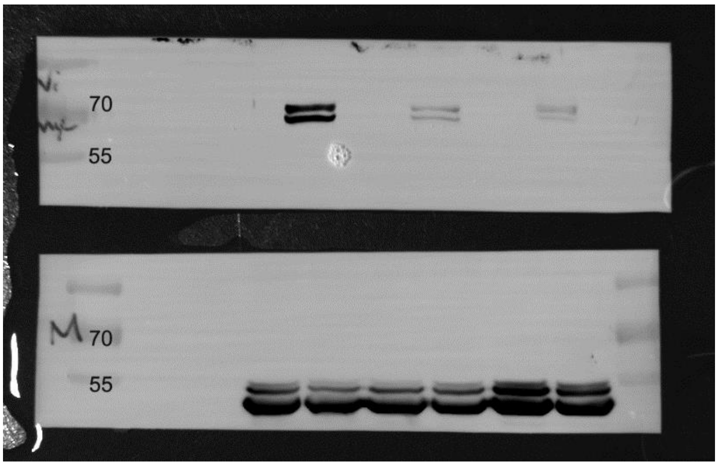

Fig. 7a

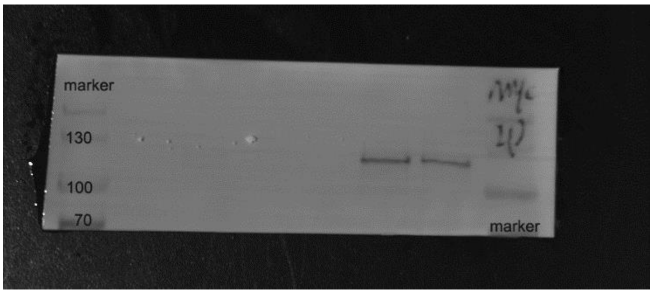

Fig. 7b

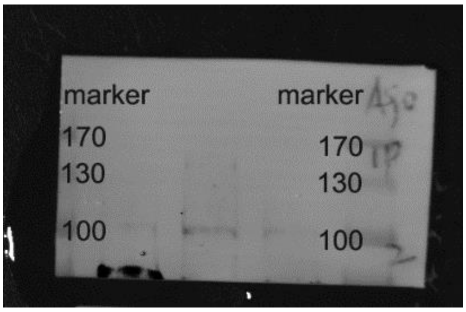

Fig. 7d

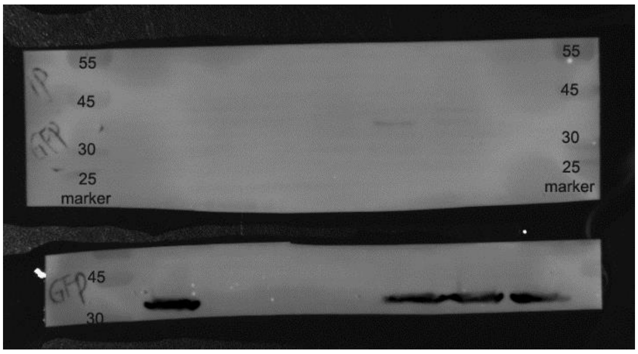

Fig. 7g

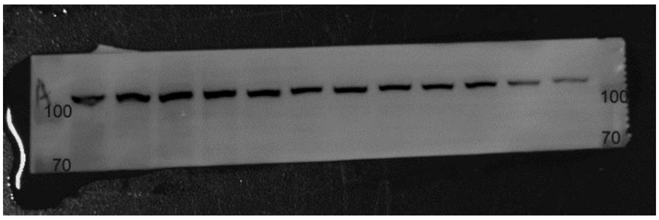

Fig. 8a

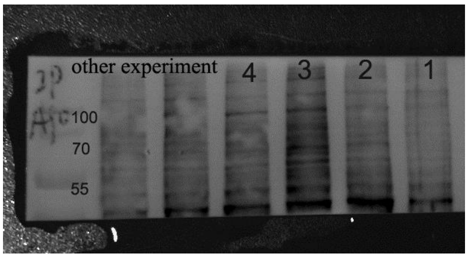

Fig. 8b

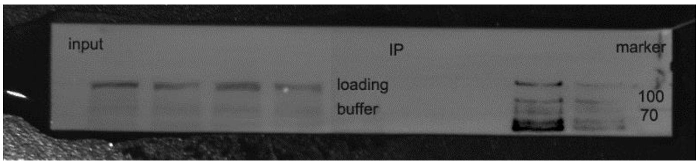

Supplementary Table 1. Putative SUMOylation sites on TARBP2. SUMO-sites of human TARBP2 protein were predicted by the program of Abgent SUMOplot (http://www.abgent.com/sumoplot/). K^{52} is the highest score (>0.9) in all possible SUMO-sites. We demonstrated K^{52} is a true SUMO acceptor site. This is related to Fig. 2.

No.	Pos.	Group	Score
1	K52	PVYDL LKAE GQAHQ	0.91
2	K96	VALKH LKGG SMLEP	0.73
3	K29	LAANP GKTP ISLLQ	0.57
4	K44	YGTRI GKTP VYDLL	0.57
5	K190	SGPAH RKEF TMTCR	TMTCR0.09

Supplementary Table 2. The sequences of shRNA and miRNA primers used in PCR

**shRNAs**		
Senp1sh1	Fwd.	ccggtgggaacattcagtacatgactcgagtcatgtactgaatgttcccttttg
	Rev.	aattcaaaaagggaacattcagtacatgactcgagtcatgtactgaatgttccca
Senp1sh2	Fwd.	ccggtagaatactcttgcaataccctcgagggtattgcaagagtattcttttttg
	Rev.	aattcaaaaaagaatactcttgcaataccctcgagggtattgcaagagtattcta
SUMO1sh	Fwd.	ccggtccttcatattaccctctccttctcgagaaggagagggtaatatgaaggtttttg
	Rev.	aattcaaaaaccttcatattaccctctccttctcgagaaggagagggtaatatgaagga
TARBP2sh	Fwd.	ccggtctcatggatgtgcaccctttgctcgagcaaagggtgcacatccatgagtttttg
	Rev.	aattcaaaaactcatggatgtgcaccctttgctcgagcaaagggtgcacatccatgaga
**qRT-PCR primers**		
miR21	RT	gtcgtatccagtgcagggtccgaggtattcgcactggatacgactcaaca
	q-PCR	gcccgctagcttatcagactgatg
miR10b	RT	gtcgtatccagtgcagggtccgaggtattcgcactggatacgaccacaaa
	q-PCR	gcgccgtaccctgtagaaccgaa
miR19b-3p	RT	gtcgtatccagtgcagggtccgaggtattcgcactggatacgactcagtt
	q-PCR	gccgctgtgcaaatccatg
miR27ac	RT	gtcgtatccagtgcagggtccgaggtattcgcactggatacgactgctca
	q-PCR	gcctgagggcttagctgcttg
miR125b-1	RT	gtcgtatccagtgcagggtccgaggtattcgcactggatacgactcacaa
	q-PCR	gcctatccctgagaccctaa
miR146	RT	gtcgtatccagtgcagggtccgaggtattcgcactggatacgacaaccca
	q-PCR	gcctgtgagaactgaattcc
miR296-3p	RT	gtcgtatccagtgcagggtccgaggtattcgcactggatacgacggagag

	q-PCR	caatcgagggttgggtggag
miR331-3p	RT	gtcgtatccagtgcagggtccgaggtattcgcactggatacgacttctag
	q-PCR	gcctggcccctgggcctatc
let7a-1	RT	gtcgtatccagtgcagggtccgaggtattcgcactggatacgacaacta
	q-PCR	gccgctgaggtagtaggttgta
let7c	RT	gtcgtatccagtgcagggtccgaggtattcgcactggatacgacaaccat
	q-PCR	gccgctgaggtagtaggttgta
reverse		gtgcagggtccgaggt
U6	Fwd.	cgcttcggcagcacatatac
	Rev.	aggggccatgctaatcttct
GAPDH	Fwd.	ctcaagggcatcctgggcta
	Rev.	atgaggtccaccaccctgtt
pre-miR125b-1	Fwd.	cagtccctgagaccctaacttg
	Rev.	agagcctaacccgtggattt
pre-miR30a	Fwd.	cgctgtaaacatcctcgac
	Rev.	catccgactgaaagcccatc
pre-miR21	Fwd.	cattgtgggttttgaaaaggtta
	Rev.	ccacgactagaggctgacttaga
pri-miR21	Fwd.	taccatcgtgacatctcca
	Rev.	cagacagaaggaccagagtt
pri-miR130b	Fwd.	tcagatccctgcagaccaccctg
	Rev.	ggcagcaagctccctttcccc
